# Dynamic mechanical response and crack evolution law of raw coal loaded by dynamic-static coupling under three-dimensional constraints

**DOI:** 10.1038/s41598-024-59135-y

**Published:** 2024-04-15

**Authors:** ShunKun Zhao, ShanYang Wei, Lin Zhang, Xianggui Tian, XingZhuan Yang, Xing Wang

**Affiliations:** 1 School of Mines, Guizhou University, Guizhou, 522100 China; 2 Guizhou Panjiang Coal & Electricity Group Technology Research Institute Co., Guizhou, 522100 China; 3 Bijie Zhongcheng Energy Co. LTD, Guizhou, 553308 China

**Keywords:** Deep coal rock, Three-dimensional dynamic and static combination, SHPB, Dynamic mechanical properties, HJC constitutive model, Civil engineering, Coal, Hydrogeology, Mineralogy

## Abstract

This paper presents the investigation of the dynamic mechanical properties of coal rock under complex stress conditions at depth, based on the improved Separate Hopkinson Pressure Bar Test System. A total of 15 groups of coal samples were used to perform dynamic impact tests under different conditions. The changing rules of dynamic strength, crushing, fractal dimension and damage modes of coal under different stress conditions were analyzed. A total of nine groups of coal samples were selected for numerical simulation using ANSYS/LS-DYNA. The results show that: (1) The stress–strain curves of coal specimens under different strain rates, different confining pressures and axial pressures have basically the same trend and the curves show a certain jump forward. (2) The peak dynamic stress of the coal specimens increased linearly with the increase of strain rate and confining pressure, and the ambient pressure limited the expansion of internal cracks of the coal specimens under impact loading. Based on the experimental and simulated data, the maximum relative errors between the experimental and simulated data were determined to be 2.9578% for Group A, 6.177% for Group B, and 6.382% for Group C, respectively. (3) The damage modes of the coal samples under the three-dimensional dynamic-static combined loading were mainly “X” type and “conical” shear damage. The fractal dimension increases with the increase of strain rate, decreases with the increase of confining pressure, and first decreases and then increases with the increase of axial pressure. This research achievement can provide theoretical support for the prevention of dynamic disasters in deep coal mine engineering.

## Introduction

With the continuous development of the mining of mineral resources from shallow to deep, the exploitation and utilization of mineral resources and energy at depth has become an inevitable trend. According to statistical data, the depth of coal, geothermal, non-ferrous metal and oil mining is over 1500 m, 3000 m, 4350 m and 7500 m respectively^1^. At the same time, as the world's population increases, so does the need for space for infrastructure development, so the importance of underground space continues to grow. As the operating depth increases, the operating conditions become more and more difficult, so that one is confronted with a complex technical environment, such as high geo-stress, high geothermal temperature, increased shock loads and increased water shocks^2–4^. As a result, energy disasters often occur in the extraction of mineral, oil and gas deposits at depth and extraction is becoming increasingly difficult.

However, the dynamic mechanical properties of materials differ considerably from the static mechanical properties. To study the mechanical properties of materials under impact loading, Kolsky invented the Split Hopkinson Press Bar (SHPB) in 1949, which is widely used in studying the mechanical properties of concrete^5^, soil, rock materials^6–9^ steel^10^ and other composite materials^11–13^ under different strain rates and loading conditions. At present, the increasing depth of mineral resources mining rock explosion, impact soil pressure and other dynamic disasters catastrophic phenomenon may occur and shallow mining conditions are obviously different, so the researchers use the improved SHPB test apparatus to perform a series of uniaxial, biaxial and triaxial loading conditions, as well as coupled with other conditions such as temperature and moisture content and other dynamic mechanical response, and has achieved significant results.

Studies have shown that the mechanical properties of shallow rock materials are very different from those of deep rock materials and that the mechanical response of deep rock is characterized by non-linearity compared to the mechanics of shallow rock^14–18^. As early as 1962, P. B. Attewell used the SHPB apparatus to study igneous and sedimentary rocks and obtained viscoelastic models for both^19^. Subsequently, Kumar^20^ analyzed the dynamic strengths of basalt and granite in the range of strain rates from 2 × 10 to 3 × 10 10 pis/s and derived the rule of variation of the dynamic mechanical strengths of basalt and granite under the coupling of strain rate and temperature. Frew et al., improved Hopkinson's compression bar system by adding a piece of copper sheet (shaper) to the end of the incident bar. A nearly constant load with strain rate was obtained and it was concluded that the dynamic modulus of elasticity of the rock does not vary with strain rate^21^. With the in-depth study of the dynamic mechanical properties of coal rock, it becomes clear to many that the one-dimensional conventional dynamic mechanical properties of coal rock can no longer meet the requirements of mineral resources. In fact, many coal rocks are in a three-dimensional stress state, so the development of conventional triaxial SHPB testing equipment has once again promoted the development of conventional dynamic mechanics of coal rock. Li and Green et al.^22,23^, who conducted triaxial dynamic SHPB experiments, concluded that the dynamic mechanical strength of granite increases with the increase of strain rate and confining pressure. The strength increases with strain rate to a relatively small extent.

When mineral resources are mined at great depths, the coal rock is dynamically loaded under static stress, resulting in a dynamic-static stress state. In the study of the dynamic mechanical properties of coal under dynamic-static combined loading, many research results show that there is an obvious influence of strain rate and peripheral pressure on the dynamic mechanical properties of rock-like materials, that is, dynamic parameters such as peak dynamic strength, elastic modulus, absorbed energy, etc. of the rock increase with the increase of strain rate and peripheral pressure^24–26^. However, as the mining of mineral resources penetrates deeper and deeper, the composition of geo-stress to which the deep coal rock is subjected changes, and when the mining depth exceeds the critical depth, the horizontal stress is lower than the vertical stress^27^. Therefore, when studying the dynamic mechanical properties of deep coal rock, not only the influence of high confining pressure on the dynamic mechanical properties of deep coal rock should be considered, but also the initial factor of axial pre-static stress. The studies of many researchers show that when the axial prestress is below the critical value, the initial value of axial prestress hinders the expansion of fractures in the coal rock, thus improving the dynamic mechanical properties such as the peak dynamic strength and elastic modulus of the coal rock. On the other hand, if the axial prestress exceeds the critical value, the axial prestress promotes the expansion of the fractures and reduces the dynamic mechanical parameters of the coal rock^28–30^.

In order to study the dynamic mechanical properties of deep coal rock, based on the research results of previous researchers, the three-axis Hopkinson Pressure Bar (SHPB) testing device is used in this paper for the three-dimensional pre-static loading and strong disturbance conditions of deep coal rock to perform the dynamic impact test on the coal specimens under the change of strain rate. The change rule of three-dimensional dynamic mechanical properties of the coal specimens under different conditions is analyzed, and the change rule of fragmentation energy and fractal dimension based on the three-dimensional dynamic and static combined loading test is discussed. Based on the three-dimensional dynamic and static combined load test, the change rules of crushing energy dissipation and fractal dimension of the coal samples were discussed. With the help of ANSYS/LS-DYNA numerical simulation software, the test process of coal samples under three-dimensional dynamic and static combined loading was simulated, and the damage process that could not be determined in the experimental process was determined to confirm the results of the test.

## Experimental device and principle

### Experimental system

**Figure 1:**
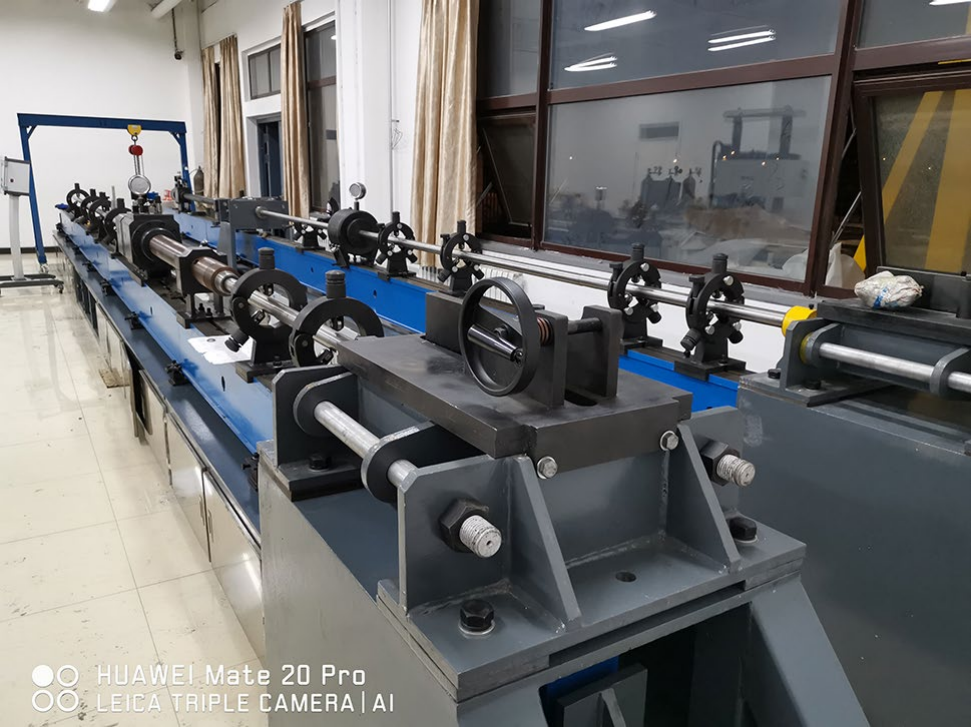
Actual diagram of the test device.

**Figure 2:**
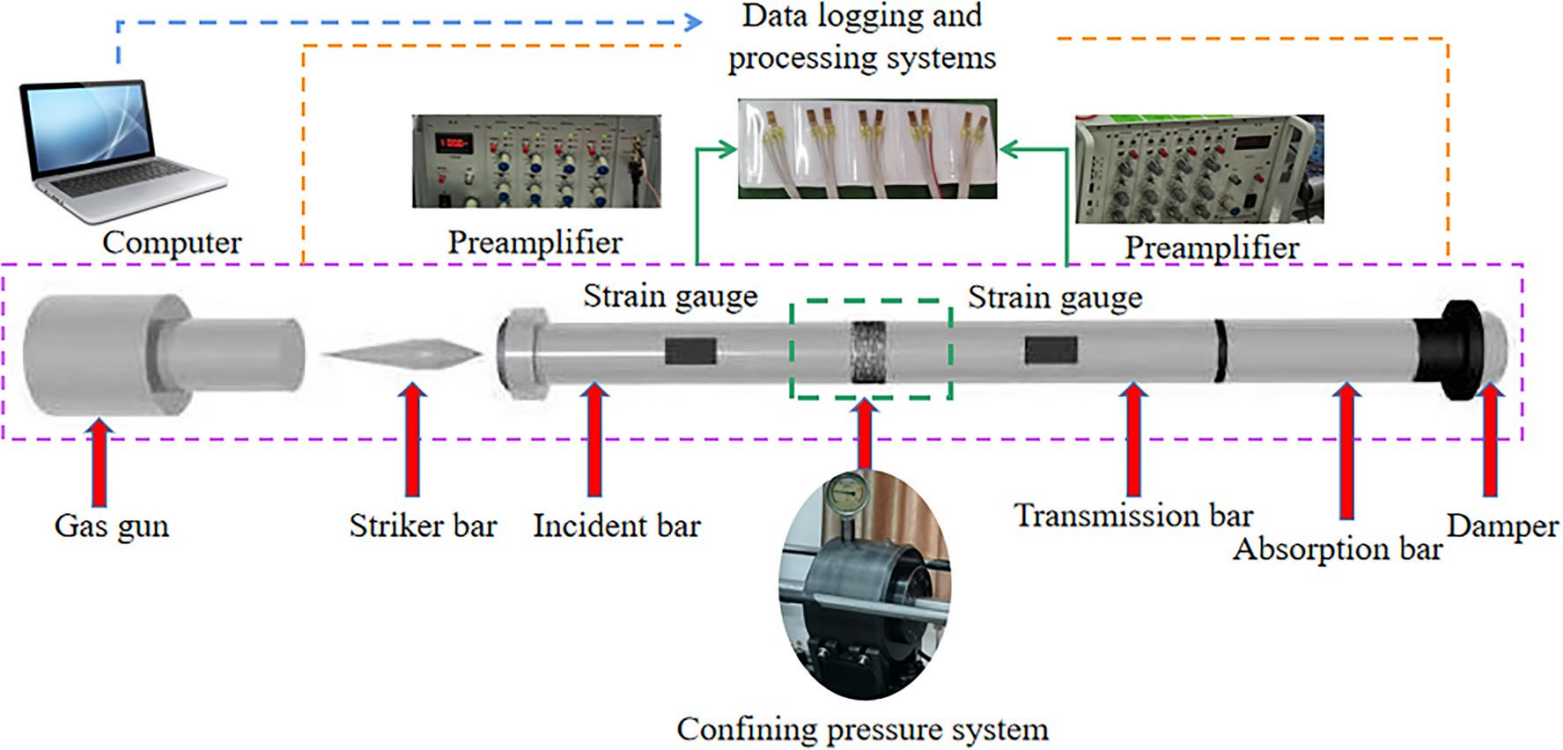
Schematic of the test setup.

In this experiment, the triaxial dynamic and static combination test system with Hopkinson bar (SHPB) of the Safety Laboratory of China University of Mining and Technology (Beijing) is used. This test system can increase the active confining pressure and axial preload compared to the conventional Hopkinson rod test system. The projectile, incident rod and reflector rod of the device are made of alloy steel with a length of 400 mm, 3000 mm and 2500 mm, the diameters are 50 mm, the modulus of elasticity is 206 GPa and the density is 7.74 g/cm^3^. The test apparatus is shown in Fig. 1 and the schematic drawing in Fig. 2.

### Sample preparation

**Figure 3:**
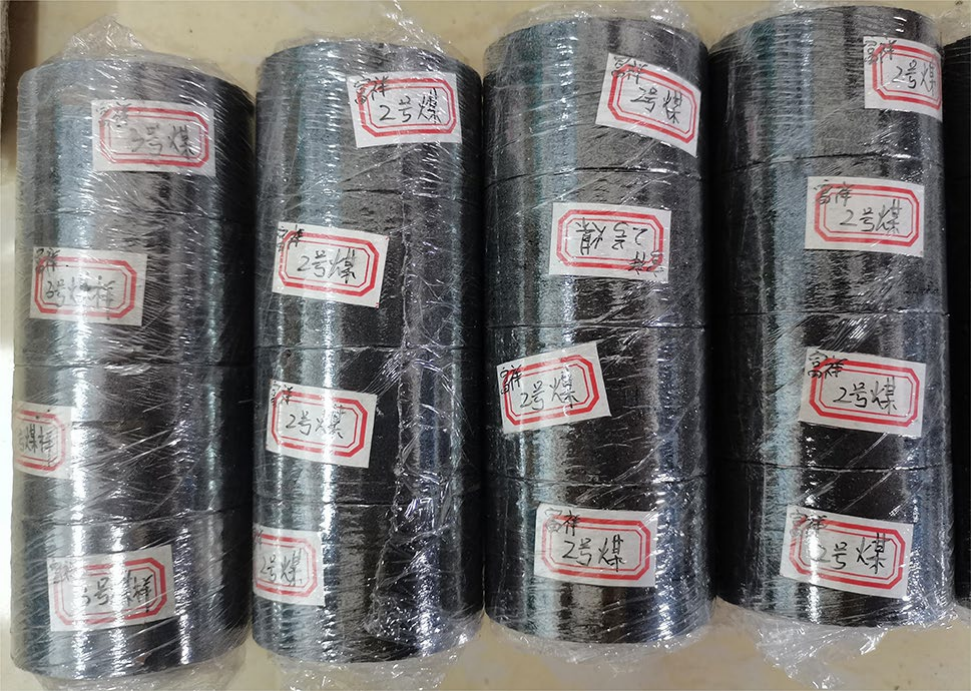
Four sets of four typical coal samples.

The coal samples were selected from Longfeng coal mine in Guizhou province, and the coal type was main coking coal. To minimize the test error, the coal samples were taken from the same homogeneous coal rock with the same depth of 400 m. The coal rock was processed into a sample with a length to diameter ratio of 0.6 to minimize the influence of the axial inertia effect. According to the requirements of the International Society for Rock Mechanics (ISRM), the flatness of the surface of the two ends of the coal sample was within 0.02 mm, and the perpendicularity of the two end faces to the axis of the sample was no more than 0.001°^31^. Finally, a cylindrical coal sample with a diameter of 50 mm and a height of 30 mm was obtained. Figure 3 shows some typical coal samples. Before the three-dimensional dynamic-static combination load test of the coal specimens, the static internal test was performed. The uniaxial compressive strength was determined to be 18 MPa, the modulus of elasticity to be 4164 MPa and the Poisson’s ratio to be 0.33.

In order to investigate the dynamic mechanical properties of coal specimens under different strain rates, different confining pressures and different axial preloads, the test was divided into three groups of 5 coal specimens each for a total of 15 coal specimens, i.e., the first group consisted of different axial preloads, same confining pressures and same impact rates; the second group consisted of different impact rates, same axial preloads and same confining pressures; and the third group consisted of different confining pressures, same axial preloads and same impact rates. In the first group, the confining pressure was 6 MPa, the impact velocity was 7.389–7.757 m/s, the axial preload force was 4MP, 6MP, 8 MPa, 10 MPa and 12 MPa, and the values of axial preload force were 22%, 33%, 44%, 55% and 66% of the static compressive strength, respectively. In the second group, the confining pressure and axial preload were 6 MPa and the impact velocity was 7.689–14.568 m/s; in the third group, the axial preload was 10 MPa and the impact velocity was 6.539–7.647 m/s and the confining pressures were 0 MPa, 4 MPa, 6 MPa, 10 MPa, 12 MPa; as the strain rate is linearly related to the impact velocity. A change in impact velocity implies a change in strain rate.

The mass of the coal samples was measured with an electronic balance with an accuracy of 0.01 g and the wave velocity of the coal samples was measured with a non-metallic ultrasonic detector. The densities of the coal samples were determined using the formula for determining the mass, height and diameter of the coal sample during sampling. The basic physical parameters of the coal samples are listed in Table 1.

**Table 1:** Basic physical parameters of coal samples.

Coal sample number	Axial compression /(MPa)	Confining pressure /(MPa)	Mass /(g)	Height /(mm)	Diameter /(mm)	Wave velocity /(m/s)	Density /(g/cm^3^)	Impact velocity (m/s)
A-1	4	6	78.977	30.15	50.25	0.96	1.322	7.647
A-2	6	6	81.138	30.21	50.43	1.20	1.345	7.389
A-3	8	6	80.256	30.19	50.24	1.20	1.342	7.757
A-4	10	6	78.144	30.13	50.28	1.18	1.307	7.563
A-5	12	6	79.714	30.12	50.36	1.16	1.329	7.736
B-1	6	6	81.240	30.21	50.28	1.63	1.355	7.689
B-2	6	6	86.744	30.3	50.31	1.28	1.441	8.015
B-3	6	6	80.37	30.23	50.30	1.18	1.339	9.673
B-4	6	6	79.665	30.22	50.24	1.21	1.330	11.679
B-5	6	6	85.201	30.19	50.45	1.27	1.413	14.568
C-1	0	0	86.855	29.96	50.4	1.47	1.454	6.539
C-2	10	4	80.042	30.23	50.25	1.11	1.336	7.043
C-3	10	6	78.977	30.15	50.25	0.96	1.322	7.647
C-4	10	10	78.446	29.52	50.35	1.44	1.335	7.174
C-5	10	12	79.52	30.33	50.53	1.16	1.308	7.549

### Introduction of HJC model and parameter calibration

**Figure 4:**
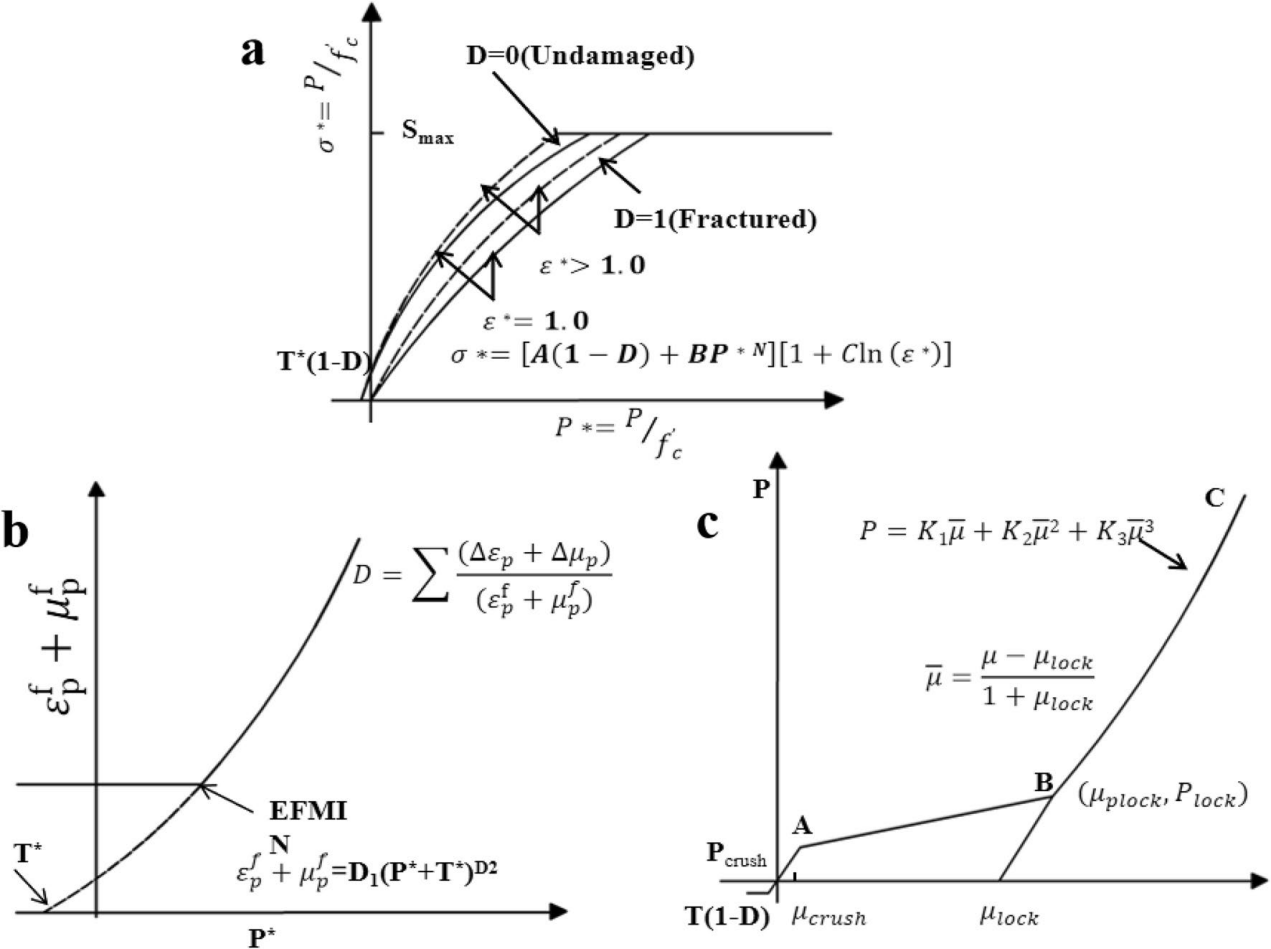
HJC model. (**a**) Equation of yield surface, (**b**) equation of damage evolution, (**c**) equation of state^33^.

The Holmquist-Johnson–Cook (HJC) model is a damage eigen structure model proposed by Holmquist et al.^32^, which is shown in Fig. 4 and mainly consists of three parts: the yield surface equation, the damage evolution equation and the state equation.

The yield surface equation is expressed as

1 $$\sigma ^{*} = \left[ {{\text{A}}(1 - {\text{D}}) + {\text{BP}}^{{{\text{*N}}}} } \right]\left( {1 + {\text{C}}\ln \dot{\varepsilon }^{*} } \right) \le {\text{S}}_{{\max }}$$

where σ^*^ is the normalized equivalent strength, σ^∗^ = σ/f_c_, σ is the true stress, f_c_ is the uniaxial compressive strength, and S_max_ is the normalized maximum equivalent yield strength. P^*^ is the normalized pressure, P^*^ = P/f_c_, P is the real pressure; ·ε^*^ is the equivalent strain rate, ·ε^*^  = ·ε/ε0, · εis the true strain rate,·ε0 is the reference strain rate; D is the damage factor (0 ≤ D ≤ 1); A is the normalized cohesive strength; B is the normalized pressure hardening coefficient; N is the pressure hardening index; C is the strain rate coefficient.

The damage evolution equation is expressed as

2 $${\text{D}} = \sum \frac{{\Delta {{\varepsilon }}_{{\text{p}}} + \Delta {{\upmu }}_{{\text{P}}} }}{{{{\varepsilon }}_{{\text{P}}}^{{\text{f}}} + {{\upmu }}_{{\text{p}}}^{{\text{f}}} }} = \sum \frac{{\Delta {{\varepsilon }}_{{\text{p}}} + \Delta {{\upmu }}_{{\text{p}}} }}{{{\text{D}}_{{\text{1}}} \left( {{\text{P}}^{{\text{*}}} {\text{ + T}}^{{\text{*}}} } \right)^{{{\text{D}}_{{\text{2}}} }} }} \ge {\text{EFMIN}}$$

where Δε_P_ and Δμ_p_ are the equivalent plastic strain and volume plastic strain, respectively, during the integral cycle. ${\upvarepsilon }_{{\text{p}}}^{{\text{f}}}$ + ${\upmu }_{{\text{p}}}^{{\text{f}}}$ is the plastic strain at constant pressure P until failure. D_1_ and D_2_ are damage constants; T^*^ = T/F_c_ is the normalized maximum hydrostatic tensile pressure, where T is the maximum hydrostatic tensile pressure the material can withstand. EFMIN is a material constant used to indicate fracture due to weak tensile wave suppression.

The equation of state is expressed as

3 $${\text{p}} = \left\{ {\begin{array}{*{20}c} {{\text{K}}_{{{{\text{elastic}\upmu }}}} {\mu ,}\;\;{\text{P}} \le {\text{P}}_{{{\text{crush}}}} } \\ {\frac{{{\text{P}}_{{{\text{crush}}}} - {\text{P}}_{{{\text{lock}}}} }}{{\mu_{{{\text{crush}}}} - \mu_{{{\text{lock}}}} }}\left( {\mu - \mu_{{{\text{crush}}}} } \right){\text{ + P}}_{{{\text{crush}}}} ,\;\;{\text{P}}_{{{\text{crush}}}} < {\text{P}} < {\text{P}}_{{{\text{lock}}}} { }} \\ {K_{1} \overline{\mu } + K_{2} \overline{\mu }^{2} + K_{3} \overline{\mu }^{3} ,\;\;{\text{P}} \ge {\text{P}}_{{{\text{lock}}}} } \\ \end{array} } \right.$$

where, μ = ρ/ρ_0_−1 is the volume strain, ρ and ρ_0_ are the current density and initial density; K_elastic_ = P_crush_/μ_crush_ is the elastomer modulus, where P_crush_ and μ_crush_ are the pressure and volume strain; when the material begins plastic deformation, μ_Plock_ and P_lock_ are the volume strain and pressure respectively, and μ = (μ−μ_lock_)/(1 + μ_lock_) is the corrected volume strain when the void is completely removed from the material; μ_lock_ is the volume strain when the density ρ reaches the crystal density ρ_grain_, and K_1_, K_2_ and K_3_ are constants.

According to the research results of Larson and Anderson^34^, the HJC model is mainly sensitive to the basic mechanical parameters such as F_c_ and the limit surface parameters A, B and N. The pressure parameters P_c_, P_1_, μ_c_ and μ_l_ have some influence on the model, while the influence of the other parameters is less than 5%. Through uniaxial SHPB test and static mechanics test on the same batch of coal samples, static and dynamic parameters were obtained, as shown in Table 2. These parameters will provide a basis for the HJC model used in numerical simulations. The base unit for each parameter was cm-g-μs. Where the RO unit is g/cm^3^; F_c_, P_c_, P_L_, and T unit is Mpa; K_1_, K_2_, K_3_ and G unit is Gpa.

**Table 2:** HJC model parameters.

Parameter	Numerical value	Parameter	Numerical value	Parameter	Numerical value
F_S_	0.0	F_C_	1.8e−004	P_L_	0.008
RO	1.34	T	2.6e−04	U_L_	0.109
G	0.01565	EPSO	1.0e−05	D_1_	0.032
A	0.59	EF_MIN_	0.01	D_2_	1.00
B	1.9	SF_MAX_	11.00	K_1_	0.0438
C	0.01	P_C_	6.0e−05	K_2_	0.0607
N	0.92	U_C_	7.38e−05	K_3_	0.109

### SHPB test principle

**Figure 5:**
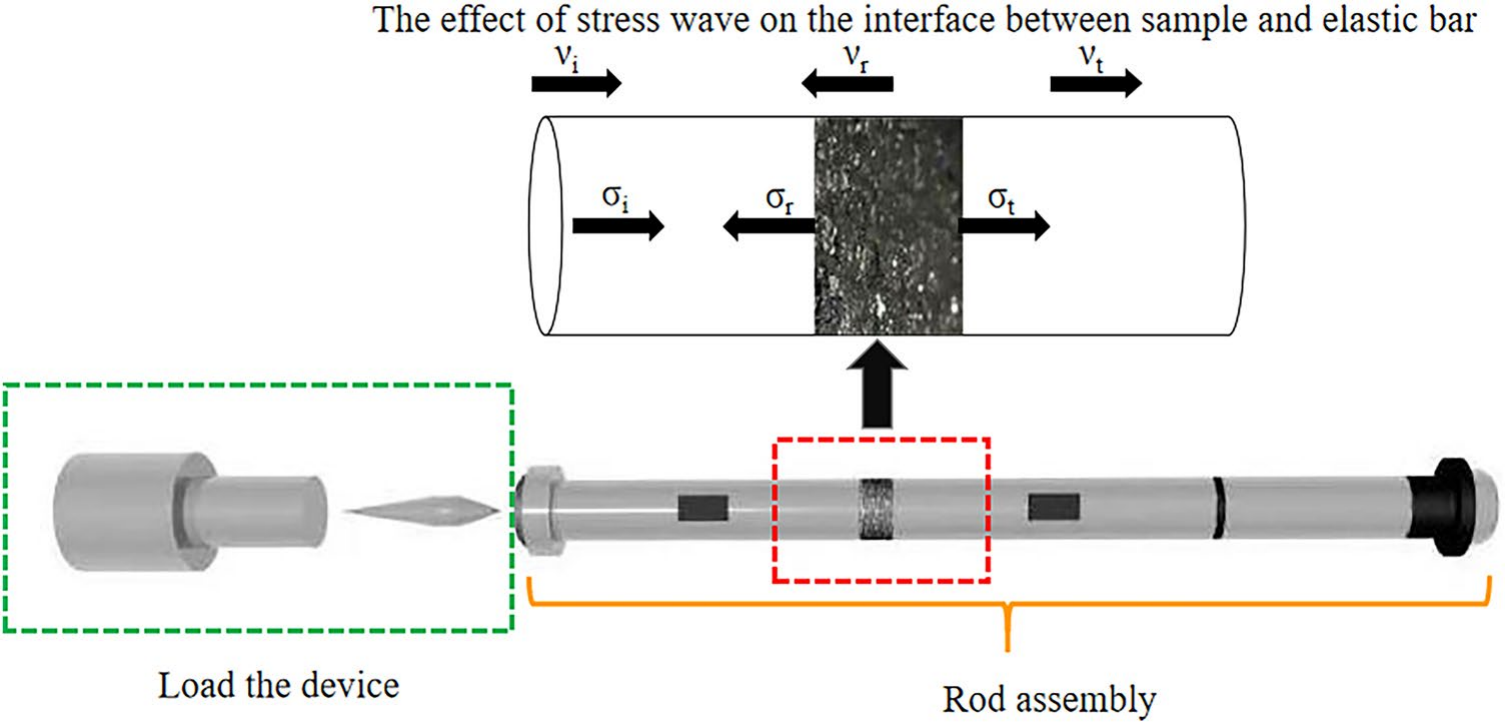
Force analysis of coal sample.

The conventional SHPB test system consists mainly of a loading device, a rod assembly and a data acquisition and recording system. As shown in Fig. 5, the impact generated by the gas canon imparts a certain velocity to the impact rod, causing the impact rod and the impacting rod to collide. Reflections and transmission waves occur up to the contact area between the impact rod and the coal and rock sample, as the wave impedance of the impact rod and the coal and rock sample are different. The transmission waves propagate further in the coal and rock sample until transmission and reflection waves occur again at the contact surface with the transmission rod, which then propagate further in the transmission rod until they are absorbed by the absorption rod and the buffer device. The propagation of the impulse signal in the SHPB rod can be determined with the strain gage attached to the incident rod and the transmission rod and then recorded with the oscilloscope.

Referring to the force analysis in Fig. 5, the stress, strain and strain rate of coal and rock samples can be analyzed and calculated. In the figure, ν_i_, ν_r_ and ν_t_ represent the incident, reflected and transmitted velocities, respectively, and σ_i_, σ_r_, and σ_t_ represent the incident, reflected, and transmitted stresses, respectively. The average stress (σ_s_), average strain, and average strain rate ε_s_ can be calculated using the following formula.

4 $${\upsigma }_{{\text{s}}} { = }\frac{{\text{A}}}{{{\text{2A}}_{{\text{s}}} }}\left( {{\upsigma }_{{\text{i}}} {{ + \upsigma }}_{{\text{r}}} {{ + \upsigma }}_{{\text{t}}} } \right)$$

5 $${\dot{\varepsilon }}_{{\text{s}}} { = }\frac{{1}}{{{\text{2L}}_{{\text{s}}} }}\left( {{\upnu }_{{\text{t}}} - {\upnu }_{{\text{i}}} {{ + \upnu }}_{{\text{r}}} } \right)$$

6 $${\upvarepsilon }_{{\text{s}}} \left( {\text{t}} \right){ = }\mathop \int \limits_{{0}}^{{\text{t}}} {\dot{\varepsilon }}_{{\text{s}}} \left( {\text{t}} \right){\text{dt}} = \frac{{1}}{{{\text{L}}_{{\text{s}}} }}\mathop \smallint \limits_{{0}}^{{\text{t}}} \left[ {{\text{V}}_{{\text{t}}} \left( {\text{t}} \right) - {\text{V}}_{{\text{i}}} \left( {\text{t}} \right) - {\text{V}}_{{\text{r}}} {\text{(t)}}} \right]$$

where, A is the cross-sectional area of the rod, As is the cross-sectional area of the coal and rock sample, and L_s_ is the length of the coal and rock sample.

Assuming that the impact pulse in the SHPB compression bar test is consistent with the one-dimensional stress wave and uniform stress hypothesis during propagation hypothesis, σ_i_, σ_r_, σ_t_, ν_i_, ν_r_ and νt in formulas (4) and (5) can be obtained by using ε_i_, ε_r_, and ε_t_ obtained by strain gages. The relationship is as follows:

7 $${\upnu }_{{\text{i}}} {{ + \upnu }}_{{\text{r}}} = {\text{C}}_{{0}} \left( {{\upvarepsilon }_{{\text{i}}} - {\upvarepsilon }_{{\text{r}}} } \right)$$

8 $${\upnu }_{{\text{t}}} = {\text{C}}_{{0}} {\upvarepsilon }_{{\text{t}}}$$

9 $${\upsigma }_{{\text{i}}} {{ + \upsigma }}_{{\text{r}}} = {\text{E}}\left( {{\upvarepsilon }_{{\text{i}}} {{ + \upvarepsilon }}_{{\text{r}}} } \right)$$

10 $${\upsigma }_{{\text{t}}} = {{{\text E}\varepsilon }}_{{\text{t}}}$$

where: E is the modulus of elasticity of the rod; C_0_ is the wave velocity with which the shock wave propagates in the incident rod and in the reflecting rod.

Equations (7)–(10) are substituted into Eqs. (4)–(6) to obtain the relationship between stress, strain, and strain rate of coal and rock specimens.

11 $${\dot{\varepsilon }}_{{\text{s}}} { = }\frac{{{\text{C}}_{{0}} }}{{{\text{L}}_{{\text{s}}} }}\left[ {{\upvarepsilon }_{{\text{t}}} \left( {\text{t}} \right) - {\upvarepsilon }_{{\text{i}}} \left( {\text{t}} \right){{ + \upvarepsilon }}_{{\text{r}}} \left( {\text{t}} \right)} \right]$$

12 $${\upsigma }_{{\text{s}}} \left( {\text{t}} \right){ = }\frac{{{\text{AE}}}}{{{\text{2A}}_{{\text{s}}} }}\left[ {{\upvarepsilon }_{{\text{i}}} \left( {\text{t}} \right){{ + \upvarepsilon }}_{{\text{r}}} \left( {\text{t}} \right){{ + \upvarepsilon }}_{{\text{t}}} \left( {\text{t}} \right)} \right]$$

13 $${\upvarepsilon }_{{\text{s}}} \left( {\text{t}} \right) = \mathop \smallint \limits_{{0}}^{{\text{t}}} {\dot{\varepsilon }}\left( {\text{t}} \right){\text{dt}}$$

According to the law of conservation of energy, the energy W_s_ absorbed by the sample can be calculated as follows:

14 $${\text{W}}_{{\text{s}}} = {\text{W}}_{{\text{i}}} - {\text{W}}_{{\text{r}}} - {\text{W}}_{{\text{t}}}$$

The incident energy (W_i_), the reflected energy (W_r_) and the transmitted energy (W_t_) can be determined by the following formula:

15 $${\text{W}}_{{\text{i}}} { = }\frac{{\text{A}}}{{{\text{C}}_{{0}} {\uprho }_{{\text{s}}} }}\mathop \smallint \limits_{{0}}^{{\text{t}}} {\upsigma }_{{\text{i}}}^{{2}} \left( {\text{t}} \right){\text{dt}}$$

16 $${\text{W}}_{{\text{r}}} { = }\frac{{\text{A}}}{{{\text{C}}_{{0}} {\uprho }_{{\text{s}}} }}\mathop \smallint \limits_{{0}}^{{\text{t}}} {\upsigma }_{{\text{r}}}^{{2}} \left( {\text{t}} \right){\text{dt}}$$

17 $${\text{W}}_{{\text{t}}} { = }\frac{{\text{A}}}{{{\text{C}}_{{0}} {\uprho }_{{\text{s}}} }}\mathop \smallint \limits_{{0}}^{{\text{t}}} {\upsigma }_{{\text{t}}}^{{2}} \left( {\text{t}} \right){\text{dt}}$$

ρ_s_ is the density of the sample.

## Comparative analysis of test results and simulation results

### Uniaxial compression and simulation modeling

**Figure 6:**
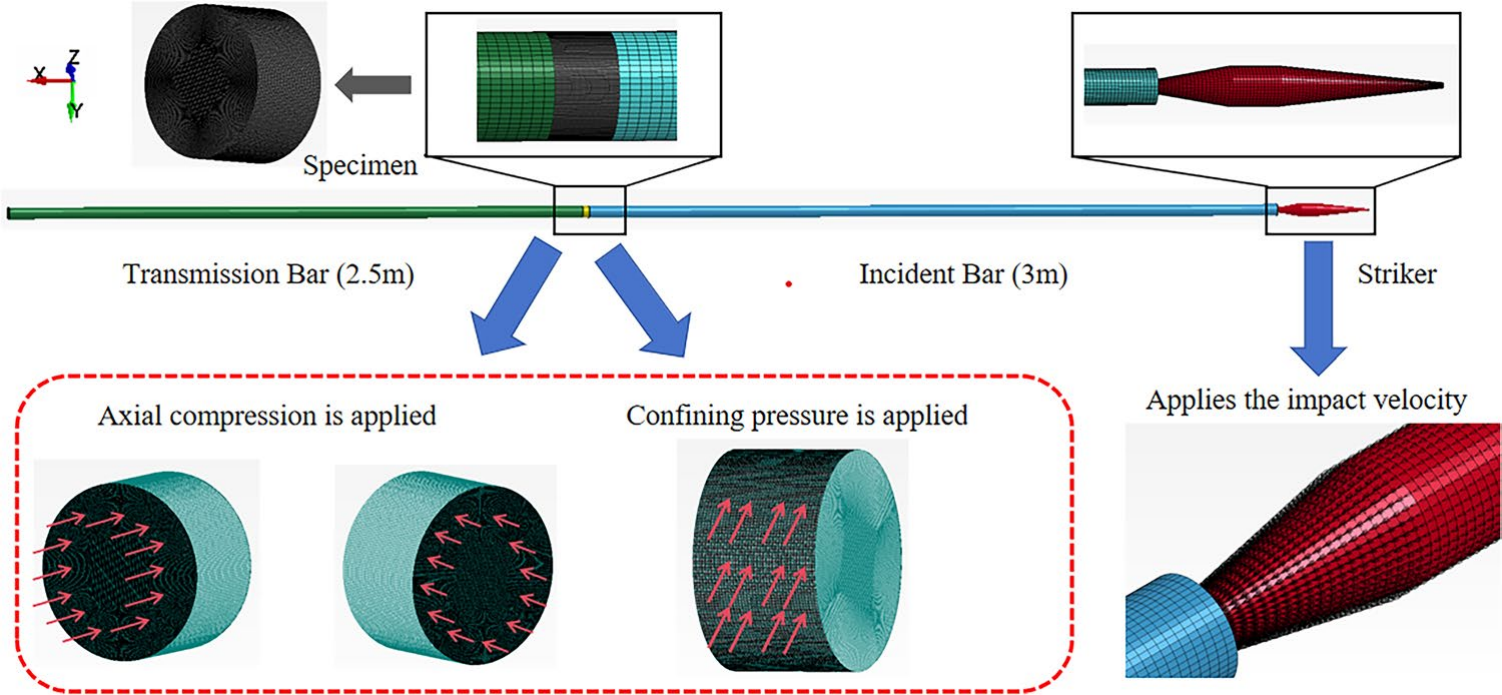
Model schematic.

Based on ANSYS/LS-DYNA, the modeling of the same size for the triaxial dynamic and static combination test system of Hopkinson bar (SHPB) is created in the safety laboratory of China University of Mining and Technology (Beijing). The model lengths of bullets, incident rods, transmissive rods and coal specimens are 400 mm, 3000 mm, 2500 mm and 30 mm, and the diameters are 50 mm, as shown in Fig. 6. A3–A5, B3–B5, C3–C5 were selected for the simulation test so that it is easy to compare and analyze the simulation results with the experimental results and see the similarities and differences between them.

**Figure 7:**
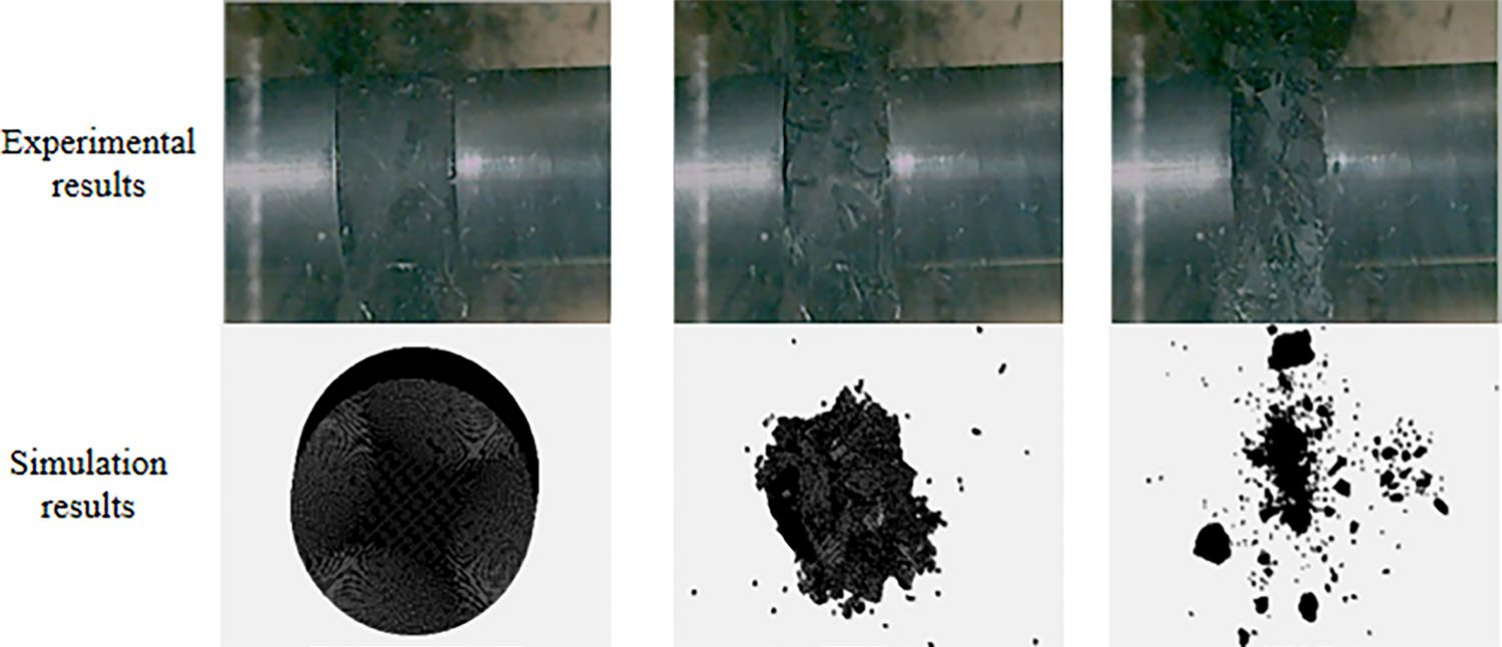
Uniaxial impact failure diagram.

**Figure 8:**
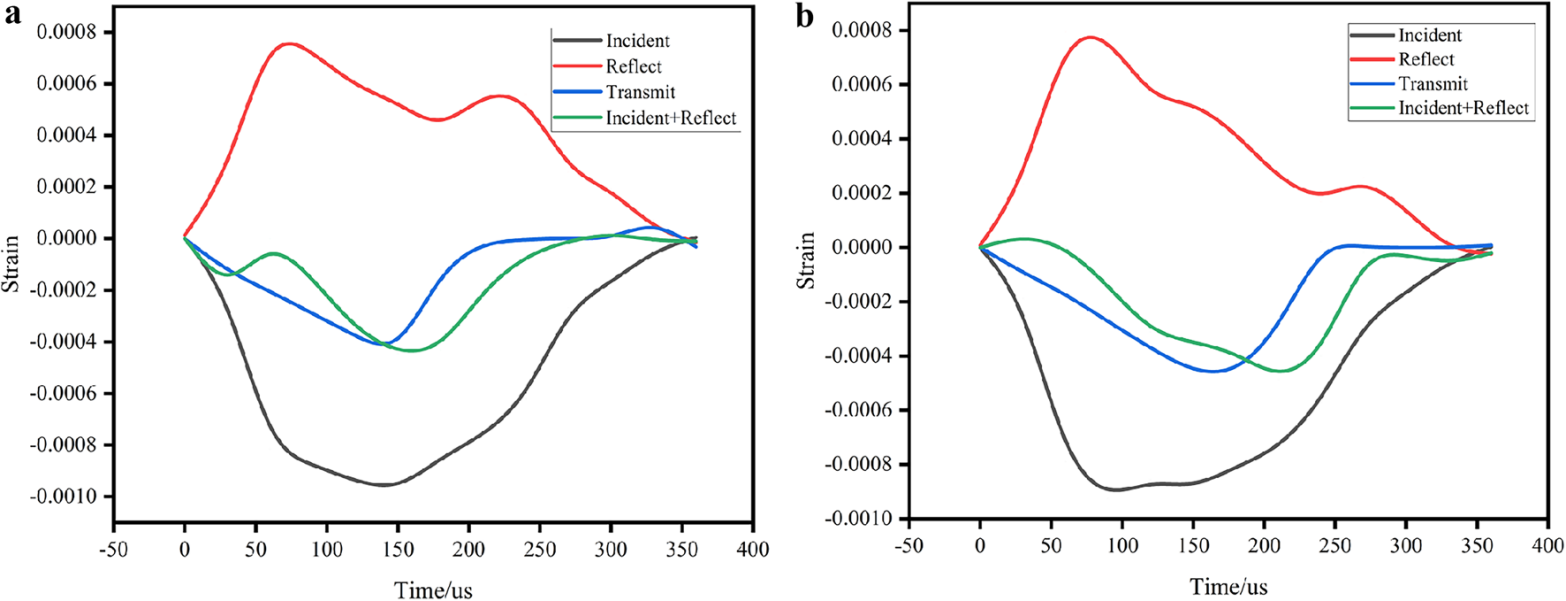
Stress uniformity test (**a**) test, (**b**) simulations.

The SHPB data processing software was used for the three-wave method to process the data in this test, and the validity of the test data was based on two assumptions: the one-dimensional stress wave and the stress uniformity. The one-dimensional stress wave assumption is mainly used to reduce the influence of the dispersion effect in the propagation of the stress wave in the calculation process. This is achieved by adding a wave shaper to the impact surface of the bullet and the incident rod and by adjusting the diameter of the incident rod^35^. To verify the accuracy of this test data, the assumption of stress uniformity is tested according to Eq. (18), i.e. the value of superposition of reflected strain and incident strain is equal to the value of transmitted strain^36^, which is verified by using uniaxial compression data of the coal specimen with uniaxial compression simulation. The test and simulation of the damage process are shown in Fig. 7, and the verification results are shown in Fig. 8.

18 $${\upvarepsilon }_{{\text{i}}} \left( {\text{t}} \right){{ + \upvarepsilon }}_{{\text{r}}} \left( {\text{t}} \right) = {\upvarepsilon }_{{\text{t}}} \left( {\text{t}} \right)$$

Figure 8 shows that the simulated waveforms and the test waveforms have some similarity and the measured and simulated values are in the same order of magnitude, but there is still some scatter between the simulated and tested data. The reason for the existence of the scatter may be caused by the operator error and the precision of the test equipment, the simulated waveforms may be different due to the definition of the rod and the sample materials. As can be seen in Fig. 7, during the uniaxial compression, the high-speed camera recorded the process of impact damage, the type of damage observed is tensile damage, which is similar to the simulated result.

### Strength characteristics of coal sample

**Figure 9:**
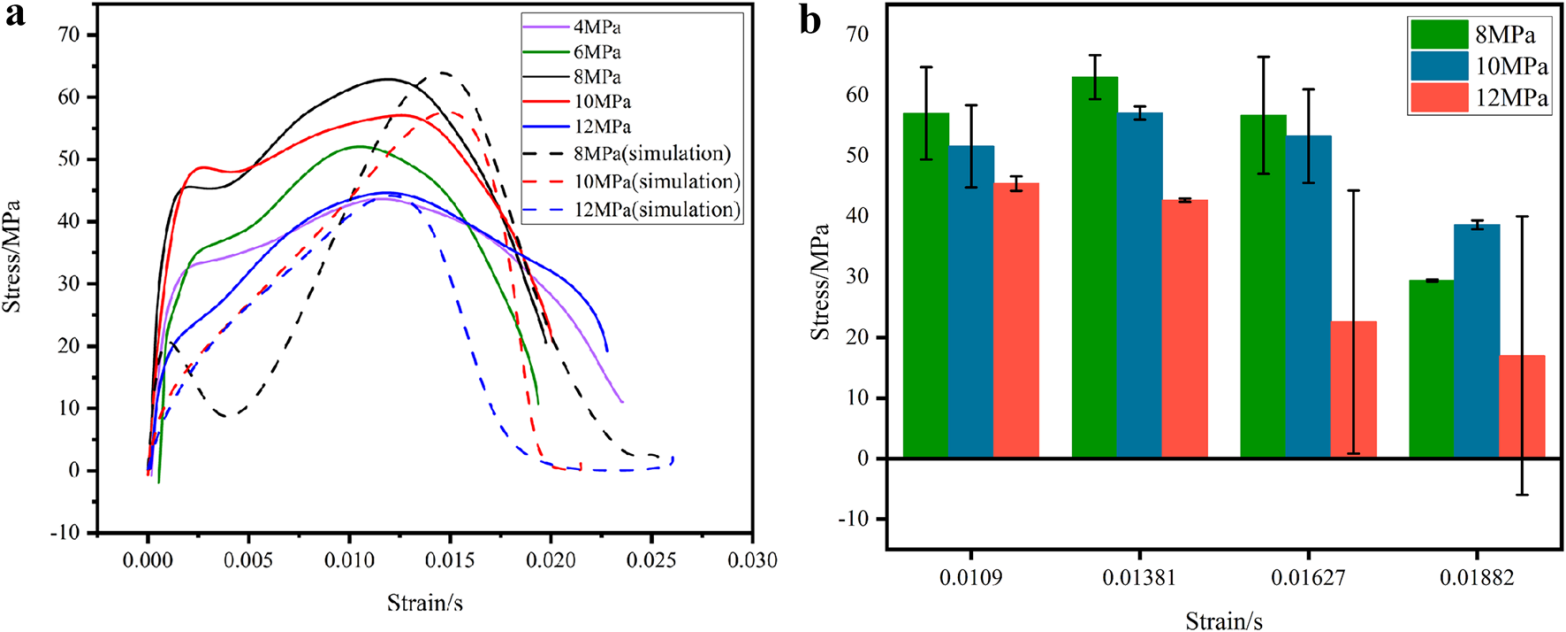
(**a**) Stress–strain of coal samples under different axial pressures. (**b**) Stress error diagram for the same strain case.

**Figure 10:**
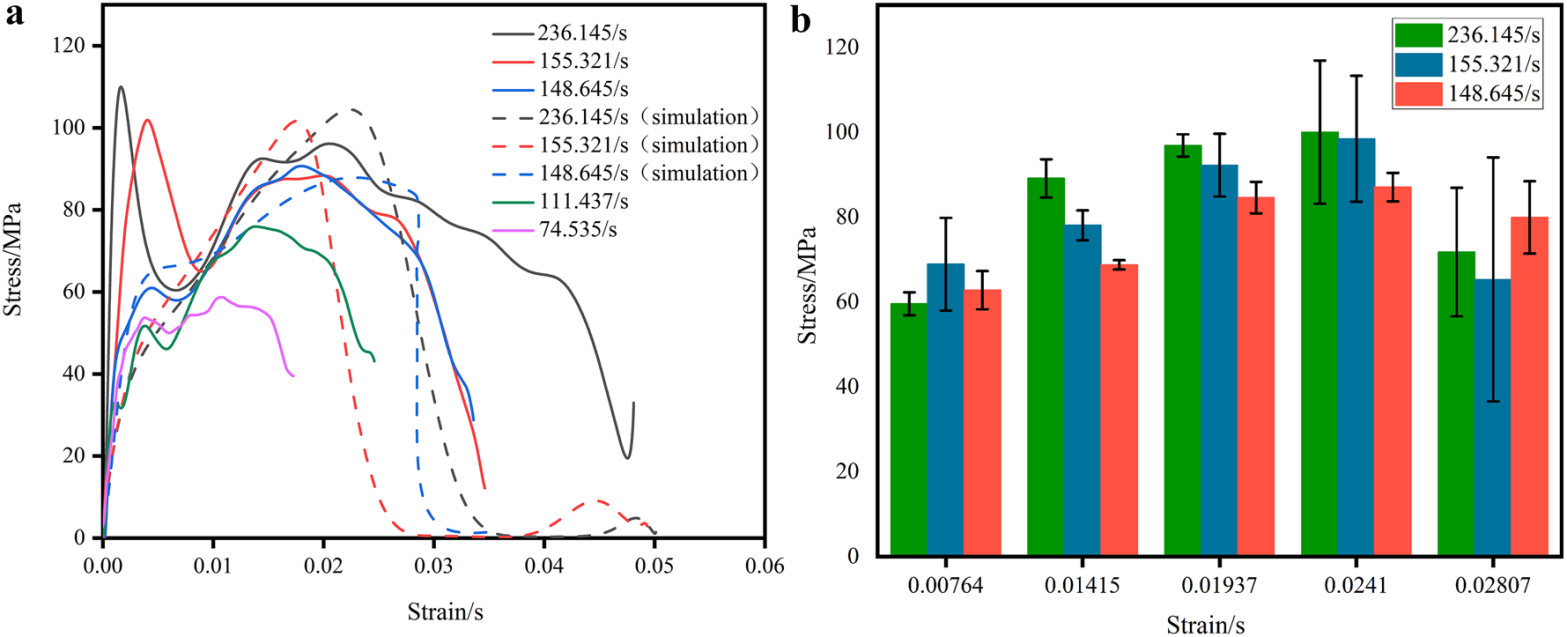
(**a**) Stress–strain of coal samples under different strain rates. (**b**) Stress error diagram for the same strain case.

**Figure 11:**
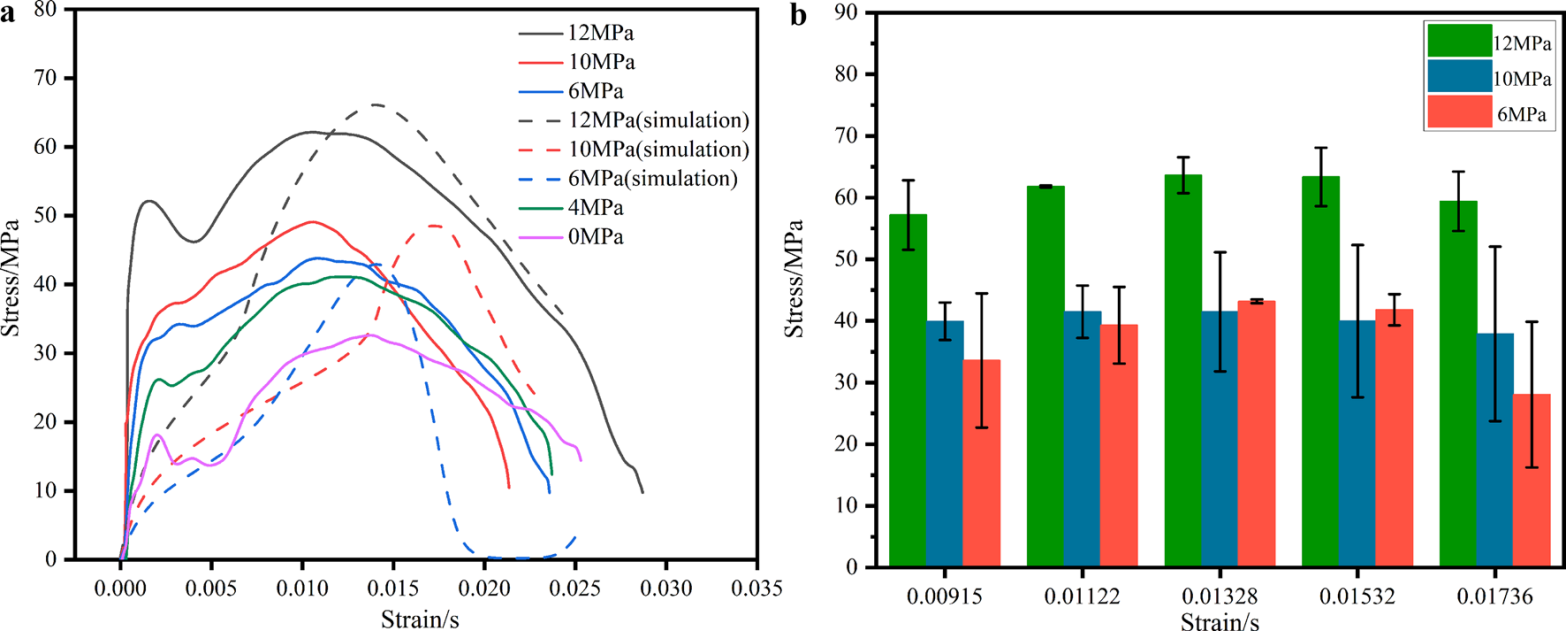
(**a**) Stress–strain of coal samples under different confining pressures. (**b**) Stress error diagram for the same strain case

Relevant studies show that the strength properties of coal rock have a significant influence on the strain rate, confining pressure and axial prestress^37,38^. Based on the experimental data and the simulation data, stress–strain curves and error bar graphs (Figs. 9, 10 and 11, where the dashed line indicates the simulation results) can be obtained for different strain rates. By analyzing and comparing the stress–strain relationship, the deformation properties of the coal under three-dimensional dynamic-static combination load can be determined together with the dynamic response behavior.

Figures 9, 10 and 11 shows that the stress–strain curves of the coal specimens at different strain rates, different confining pressures and axial pressures essentially show the same trend. By calculating and analyzing the simulation results and the experimental data, the maximum relative error is obtained (Group A: 2.9578%, Group B: 6.177%, Group C: 6.382%). This indicates that the dynamic performance of coal samples in three-dimensional impact tests can be better simulated by the construction of the HJC model. When the confining pressure and axial preload are present, the stress–strain curves are different from the impact condition (coal specimen C−1) without axial pressure at the circumference. The stress–strain curve in the state without axial circumferential pressure has an additional compression phase, and this phase of the curve is curved upwards. The reason for this phenomenon is that the rock sample is already compacted by the ambient pressure and the axial preload when the coal sample is subjected to an impact load. Therefore, the rock sample does not go through a compaction phase during the combined three-dimensional dynamic and static loading, but enters directly into the elastic deformation phase. At the same time, the phenomenon of bimodal peaks occurs at different strain rates, and different confining and axial pressures, and the curves show a certain degree of jumps, especially at different strain rates, the phenomenon of bimodal peaks is most obvious, and the reason for this phenomenon may be related to the role of charcoal in the micro-cracking of crystals, and the result is consistent with the analytical results of many scholars^22,39–41^, i.e, the stress reaches the first stress maximum (compressive strength), as the strain increases, the stress decreases, the rock sample enters the stage of yielding, and as the stress increases again, the stress reaches the second stress maximum.

Figure 9a shows the stress–strain curves of the coal specimens under different axial pressures. It can be seen from this figure that the peak stress of the coal specimens first increases with increasing axial pressure and then decreases. This is because when the axial pressure is less than the critical value, with the increase of the axial preload, the cracks of the coal specimens are continuously compressed, the dynamic compressive strength increases and the axial preload has a strengthening effect on the dynamic peak strength; when the axial pressure is greater than the critical value, with the increase of the axial preload, the dynamic peak compressive strength decreases and the axial preload promotes the crack expansion and the dynamic peak compressive strength is weakened. If the axial pressure is greater than the critical value, the dynamic peak compressive strength decreases with the increase of the axial preload, and the axial preload promotes crack expansion and weakens the dynamic peak compressive strength. In the simulation data, the dynamic peak pressure is slightly higher than in the experimental data, which is probably due to the friction that was not eliminated in the simulation. Figure 9b shows the stress error corresponding to the same strain in the experiment and simulation, and after comparison, it can be seen that under the same loading conditions, the error values before and after the peak stress of the coal samples are smaller, and the simulation results are more realistic and reliable. In the next Figs. 10b and 11b is also the same.

Figure 10 shows the stress–strain curves of coal specimens at different strain rates. It can be seen that as the strain rate increases, the peak stress of the coal specimens increases and the stress–strain curve can be divided into three phases: In the elastic phase, the stress increases in a straight line with increasing strain, and the stress–strain curve is a linear relationship; in the plastic phase, the stress increases more slowly with increasing strain, and irreversible plastic deformation of the coal sample occurs in this phase. At The end of the plastic phase, the stress reaches its maximum value. In the phase after the damage peak, when the strain continues to increase, the stress does not drop to 0. Rather, there is a certain bearing capacity due to the fact that the residual strength of the coal samples gradually increases with the increase of the confining pressure. However, since the main model of HJC does not consider the residual strength as an influencing factor, the resulting graph of simulation data shows that the stress gradually tends to 0 with the continuous increase of strain, which also indicates that the residual strength parameter should be considered when studying the main dynamic relationship of coal rock.

Figure 11 shows the stress–strain curves of coal specimens under different confining pressures. From this, it can be seen that increasing the confining pressure can improve the compressive strength of the coal specimens because the confining pressure can prevent the lateral deformation of the coal specimens during the loading process, thus increasing the compressive strength of the coal. The transition from brittle to ductile behavior occurs when the confining pressure is increased to 10 MPa. This is because coal often exhibits brittle failure characteristics at lower initial stress levels, while at higher stress levels it exhibits a gradual transition towards ductile failure characteristics. The strength variation with stress levels also exhibits nonlinearity, as shown by the red dashed line. The damage time is prolonged, and the damage process is slower compared to low confining pressure. The stress–strain changes of the coal specimens under different confining pressures have the same trend, which can be divided into three phases: elastic phase, plastic deformation phase and phase after unloading. In the simulation, there is also a similar regularity that the coal specimens gradually change from brittle gap damage to plastic flow with increasing ambient pressure, and the strain before damage also gradually increases.

**Figure 12:**
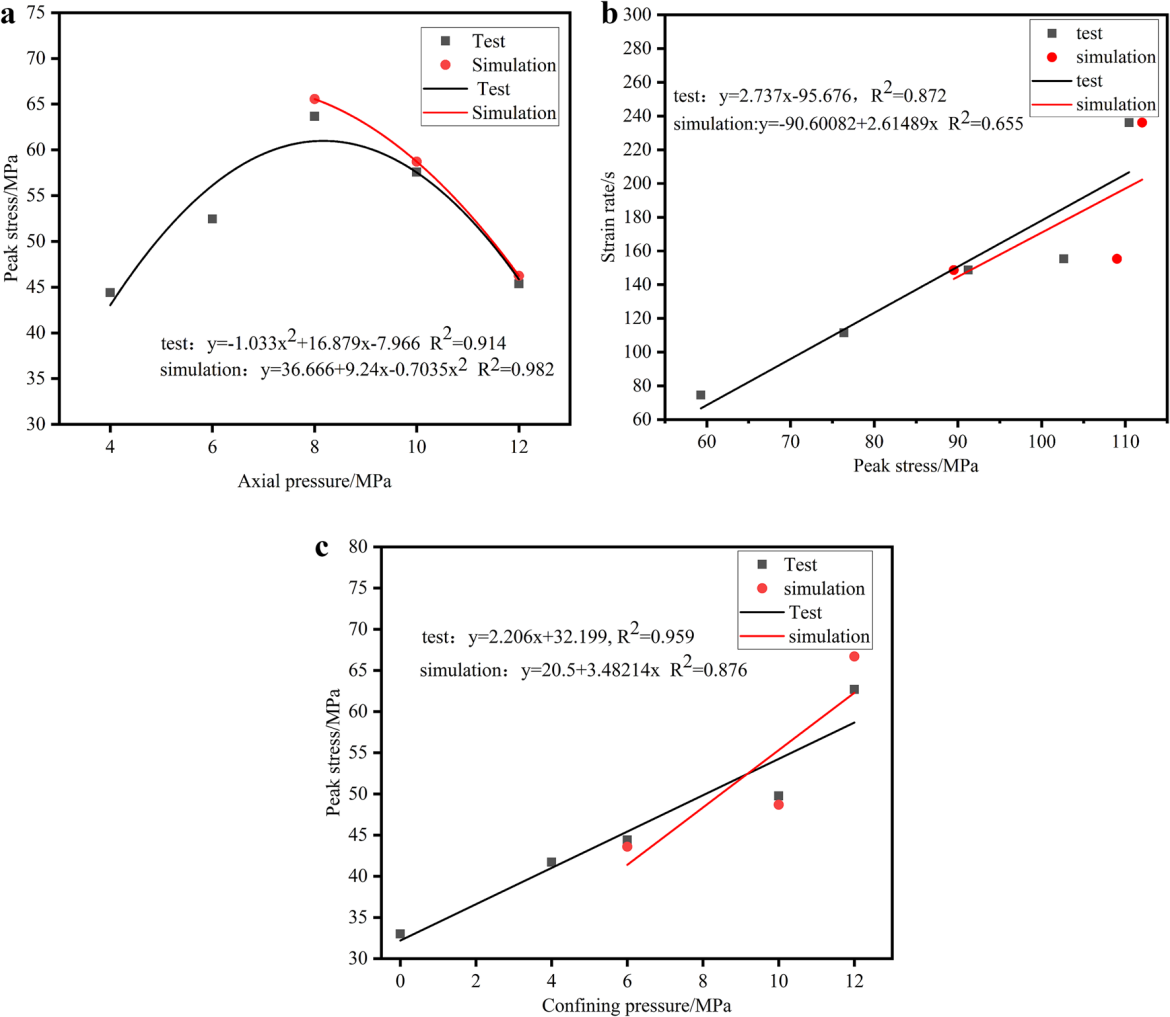
Variation curve of peak stress with axial pressure (**a**), strain rate (**b**), confining pressure (**c**).

**Figure 13:**
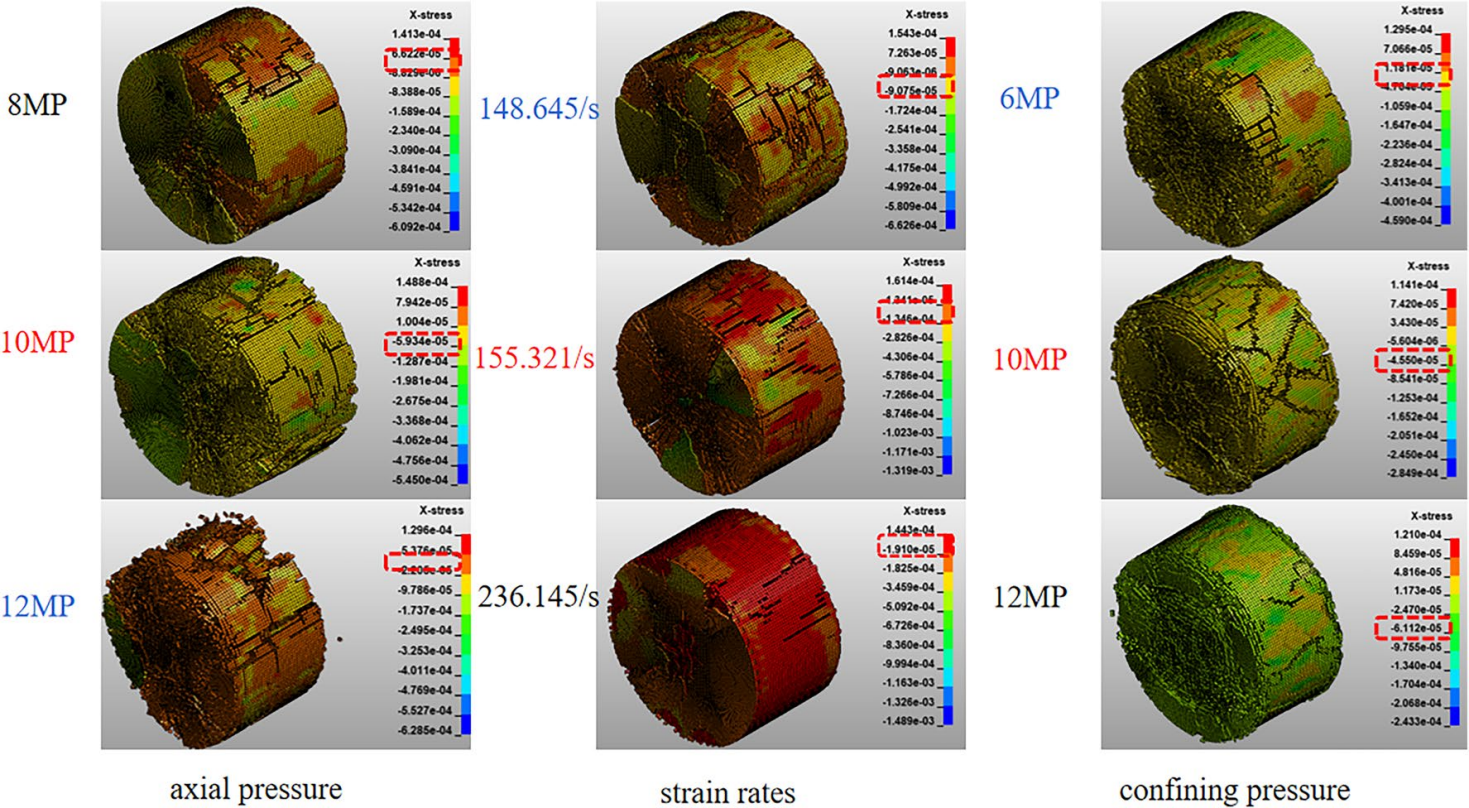
Peak stress cloud under different loading conditions.

From the plots of peak stress and stress cloud (Figs. 12 and 13) obtained from the test and simulation under different conditions, it can be seen from Fig. 12a that the dynamic peak stress of the test and simulation decreases with the increase of axial pressure, which essentially shows a single-variable quadratic equation, and the relationship of the change is as follows: y = − 1.033x^2^ + 16.879x (R^2^ = 0.914), y = 36.666 + 9.24x−0.7035x^2^ (R^2^ = 0.982). When the axial prestress is 4–8 MPa (22%σ_c_−44%σ_c_), with the increase of axial pressure, the cracks of coal specimens are continuously compressed, the dynamic compressive strength is strengthened, and the axial prestress has a strengthening effect on the peak dynamic strength. When the axial pressure is 8–12 MPa (44%σc−66%σc), the dynamic peak compressive strength decreases with the increase of axial pressure. The axial pressure promotes crack expansion and weakens the dynamic peak compressive strength, which shows that the critical axial pressure of the coal samples is 8 MPa (44% σc). From Fig. 12b, it can be seen that although the fitting accuracy of the simulated data is insufficient, the overall relationship shows the same trend as in the test, and the peak dynamic stress of the coal specimens increases linearly with the increase in strain rate. And the relationship equations are as follows: y = 2.737x−95.672 (R^2^ = 0.872), y = − 90.60082 + 2.61489x (R^2^ = 0.655).The above pattern of change is due to the fact that with the increasing strain rate, the greater the degree of coal rock fragmentation, the greater the energy to be absorbed, so the greater the dynamic peak stress of coal rock with it It can be seen from Fig. 12c that the peak dynamic stress of the coal rock in the test and simulation increases linearly with the increase of the peripheral pressure, and the relational equations are, respectively, as follows: y = 32.199 + 2.206x(R^2^ = 0.959), y = 20.5 + 3.48214x(R^2^ = 0.876). During impact loading, the ambient pressure limits the expansion of the internal cracks in the coal rock, so that the dynamic peak stress of the coal rock increases with the increase in ambient pressure during three-dimensional dynamic-static combination loading. The peak stress below 10MP may be caused by the fact that the coal specimens are in a situation where the axial pressure is uniformly loaded with the ambient pressure to cancel part of the residual stress, causing the peak stress below 10MP to change abruptly. From the above analysis, it can be seen that the dynamic compressive strength of coal rock has a significant influence on the strain rate, confining pressure and axial pressure.

### Coal rock damage mode under three-dimensional dynamic-static combination loading

**Figure 14:**
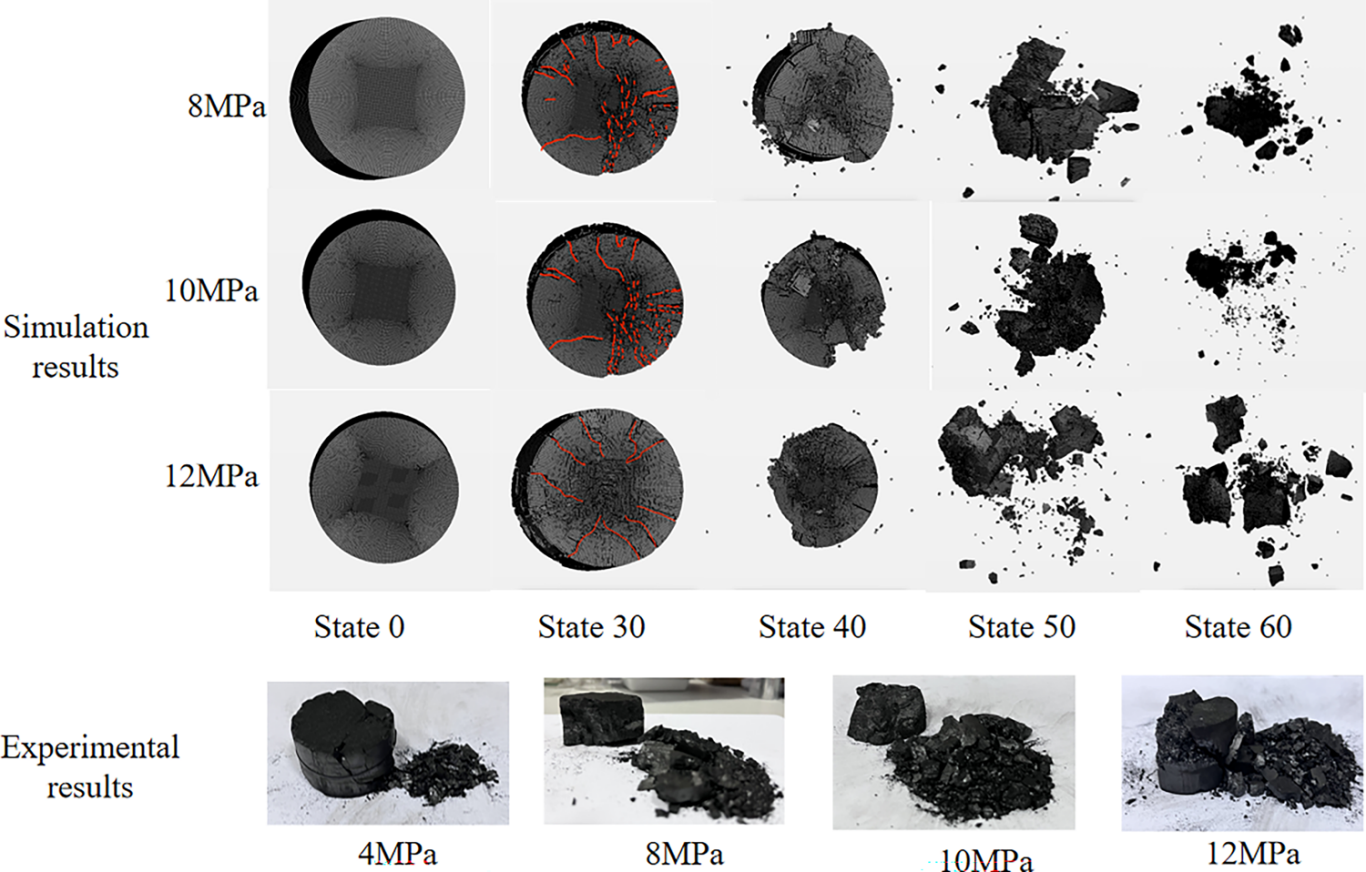
Damage pattern of coal rock under different axial pressures.

**Figure 15:**
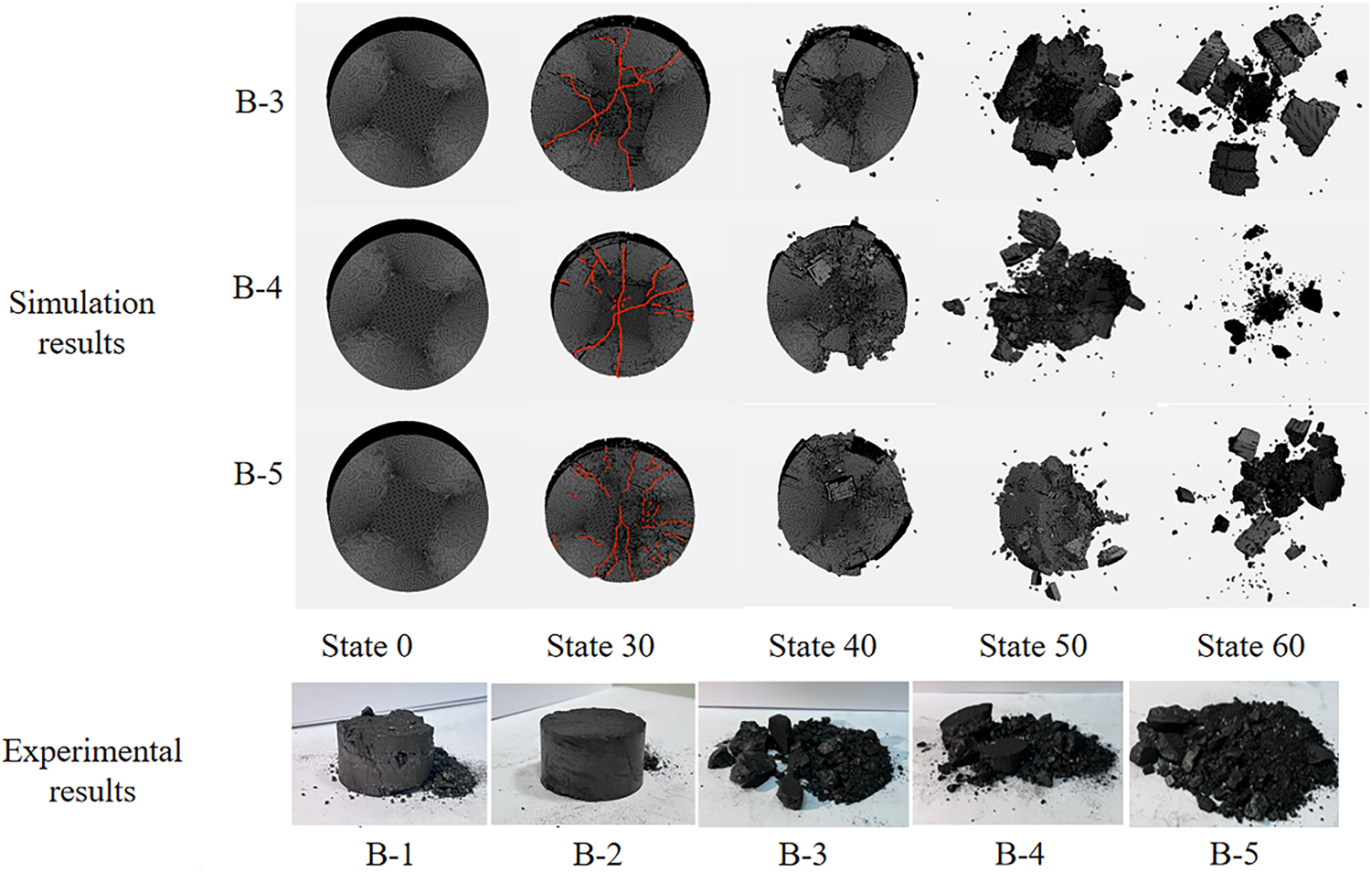
Coal rock damage pattern under different strain rates.

**Figure 16:**
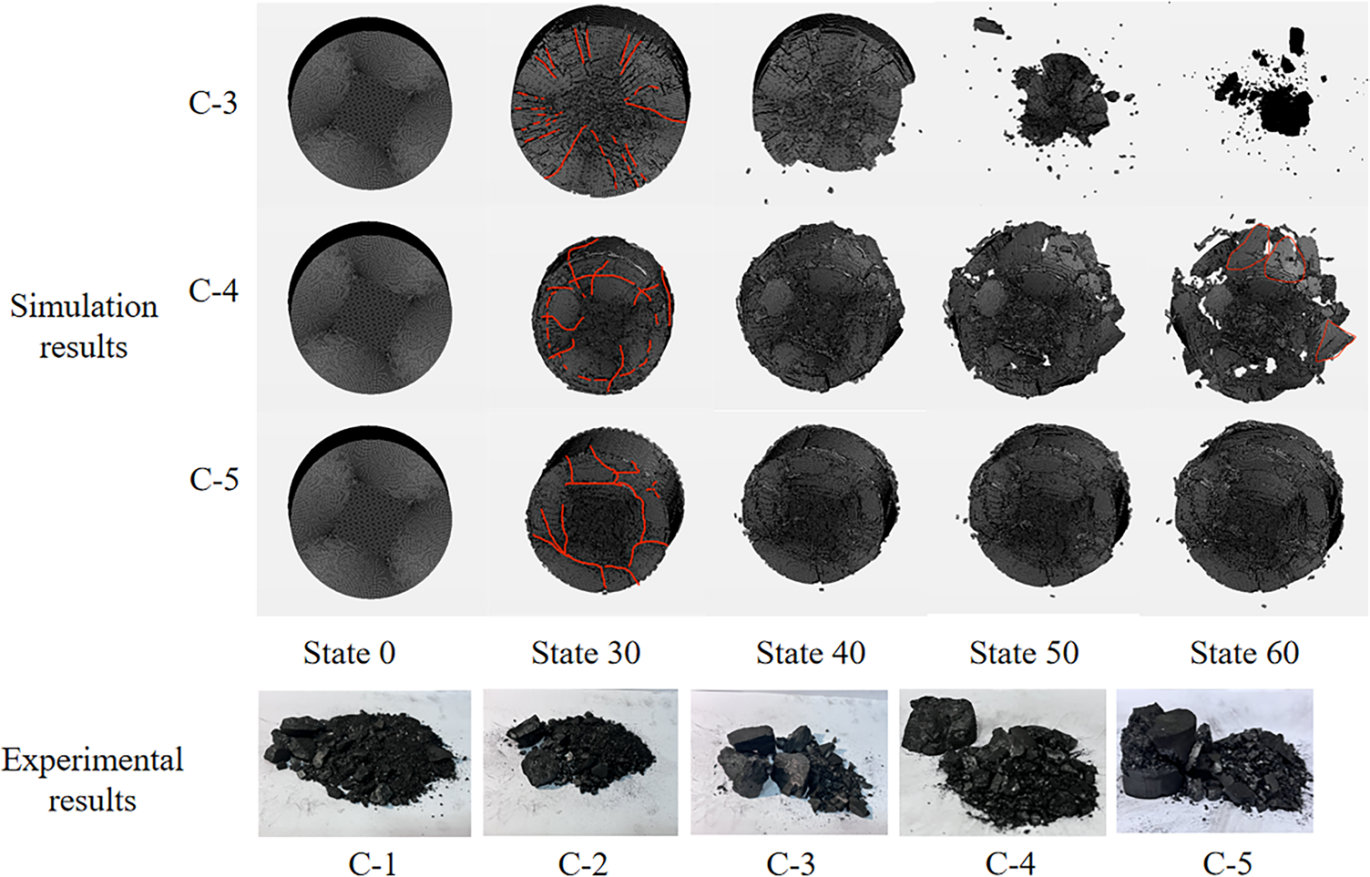
Damage pattern of coal rock under different pressures.

The damage pattern of coal rock reflects the force state of coal rock. Compared with static compression and uniaxial dynamic-static combination loading, the damage pattern of coal rock under three-dimensional dynamic-static combination loading is very different, and most of the equipment conditions of coal rock in practice are three-dimensional dynamic-static combinations. The damage patterns of coal specimens under different strain rates, different confining pressures and different axial pressure loads are analyzed, as shown in Figs. 14, 15 and 16.

#### Damage patterns of coal rock under different axial pressures

Figure 14 shows the damage modes of the coal samples under different axial pressures. It can be seen that as the axial preload increases, the degree of damage to the coal specimens first increases and then decreases and the size of the broken particles first decreases and then increases. When the axial preload is 4 MPa (22% σ_c_), the damage types of the coal specimens are mainly shear, and the degree of fragility is lower because the axial pressure to which the coal specimens are subjected is relatively low and the absorbed energy is also low. From the simulated damage history (stage 30, which is one of the stages in the simulation process of shock loading after setting the time step), it can be seen that at an axial preload of 8 MPa (44%σ_c_) and 10 MPa (55%σ_c_), the main damage mode of the coal sample is a mixture of shear and tensile damage. At this point, the coal sample is subjected to greater relative axial pressure, resulting in greater strain energy stored in the coal sample. When the coal sample is subjected to an impact load, the damage starts from the outer circumference of the coal sample. When the coal sample is subjected to an impact load, the damage starts from the outer circumference of the coal sample and the degree of destruction is greater. When the axial preload is 12 MPa (66%σ_c_), the main damage mode of coal sample is a mixture of shear and tensile in nature, Although the axial prestress is the largest at this time, but due to the coal sample of the load absorbed energy is more concentrated, from the middle part of the sample began to destroy the sample broken block size becomes larger, then the final destruction of coal samples to reduce the degree.

#### Damage patterns of coal rock at different strain rates

Figure 15 shows the damage pattern of the coal specimens at different strain rates. As can be seen from B-1, under the initial conditions of peripheral and axial pressure, the coal specimens were not severely damaged due to the low strain rate, but there are obvious shear cracks along the impact direction and a small amount of coal dust is detached from the upper and lower ends. The phenomenon described above is due to the presence of confining and axial pressure, which causes stress concentration at both ends of the coal sample. The damage pattern of this coal sample shows that the deep surrounding rock not only needs a strong prestress as initial conditions due to the impact load of the rock explosion phenomenon, but also needs a certain impact load for the rock explosion to occur. In B-2, it can be observed that with the increase in strain rate, there is a significant detachment of coal samples occurring at both ends. Additionally, cracks are present in the upper part of the coal samples, along with confining cracks and cracks aligned with the impact direction. In the simulated damage process from B-3 to B-5, due to the excessively high strain rate, the coal samples were crushed to a greater extent. As the strain rate increased, the particle size of the crushed coal samples decreased. The damage pattern is almost always in the form of “X” damage and is caused by shear damage. This form of destruction is consistent with the results of Yan et al.^42^.

#### Damage modes of coal rock under different confining pressures

Figure 16 shows the damage patterns of the coal specimens at different confining pressures. It can be seen that the degree of damage of the coal samples decreases with the increase of confining pressure, indicating that the confining pressure can largely prevent the deformation of the coal samples in the confining direction, and the higher the confining pressure is, the lower the fragmentation degree of the coal samples is and the larger the fragmentation particle size is. When the confining pressure is 0 MPa, the coal sample is subjected to a uniaxial impact load. Test C-1 shows that the coal sample is almost completely broken, and it can be seen from the only surviving pieces of coal that the coal sample suffers shear damage under the uniaxial impact load. When the confining pressure is 4 MPa, the effect of deformation restraint of the coal specimens in the annulus is relatively small due to the relatively low confining pressure. Combined with the relatively large impact force, the degree of crushing of the coal samples is higher, but compared with the uniaxial impact, the broken coal pieces are relatively large, and the shear damage of the coal samples can be seen in Experiment C-2. In the simulation, the broken pieces of the coal samples at an edge pressure of 10 MPa (C-4) are smaller due to the relatively high confining pressure. It can be seen from the edge of the broken coal samples that the direction of the broken grains of the coal samples is in the direction of the impact and that there are “conical” pieces of coal. It can be assumed that under the three-dimensional dynamic-static combination load with relatively large circumferential pressure, the micro-fracture is suppressed when the coal rock is crushed with increasing circumferential pressure. As a result, the brittle coal samples show ductile properties, and the crushing degree of the coal samples becomes lower. The damage mode of the coal samples is a mixture of shear and tensile damage. At a confining pressure of 12 MPa (C-5), the degree of comminution of the coal specimens is relatively low, and only partial detachment by shear occurs.

### Coal rock crushing block size fractal characteristics

**Figure 17:**
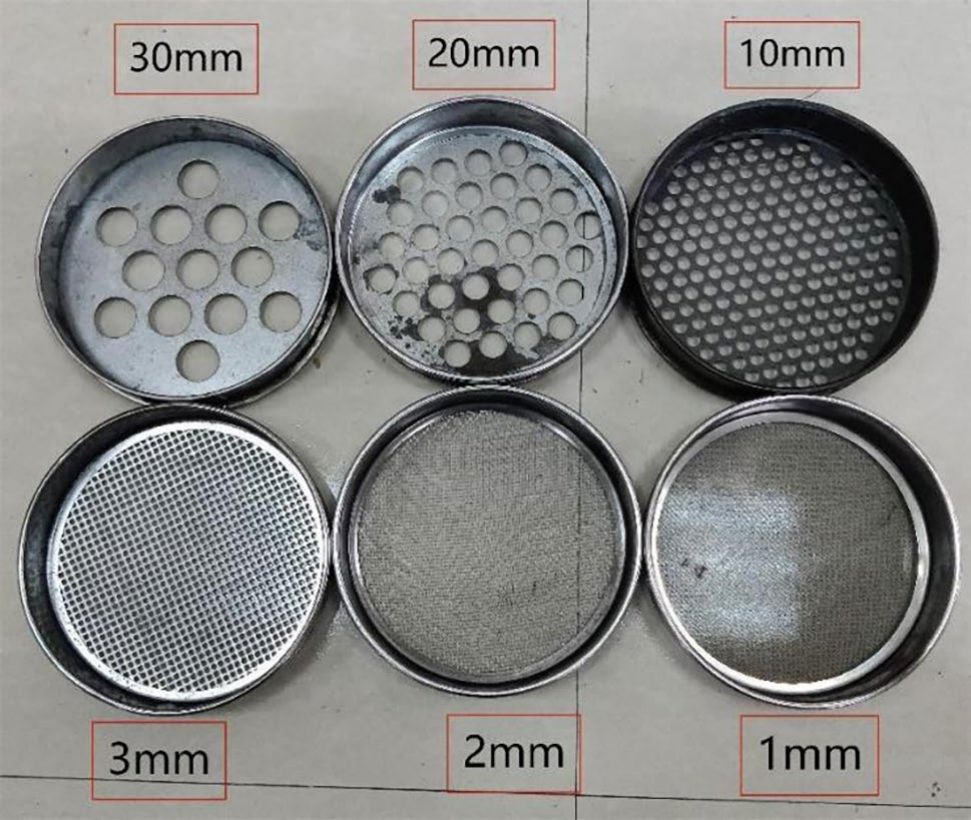
Round hole coal screen.

**Figure 18:**
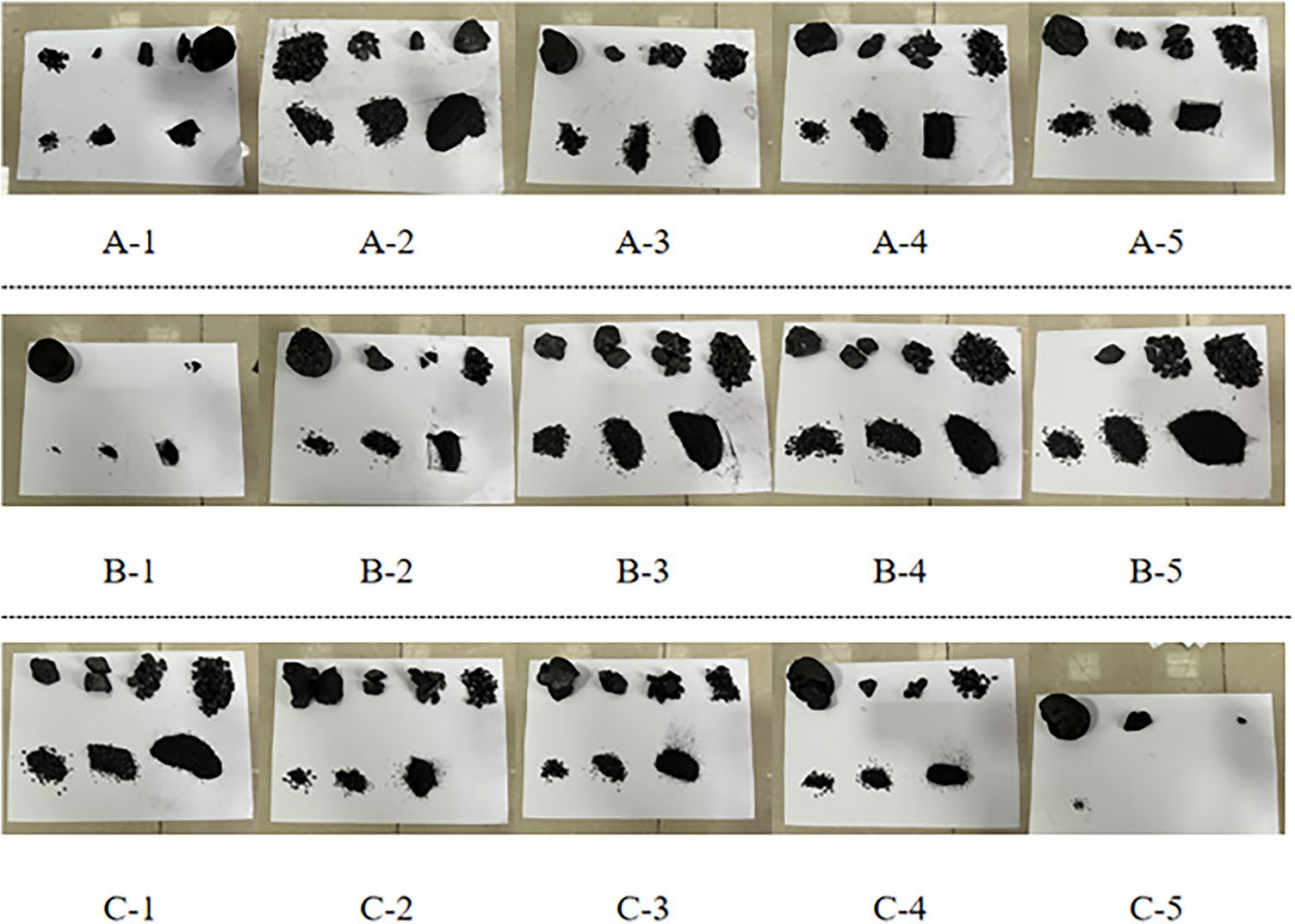
Fractal screening results under different loading conditions.

The crushing effect of coal specimens under three-dimensional dynamic and static combined loading reflects the force state of coal specimens, and through the properties of fractal theory, the crushing block size distribution can be used, to evaluate the crushing effect of coal rock, and in the previous studies, the statistical function of crushing block size distribution with R–R (Rosin–Rammler) distribution and G-G-S (Gate-Gaudin-Schuhmann) distribution^43–45^ has been used relatively frequently. In this paper, the G-G-S distribution function is used to fracture the coal samples into dimensions. From the literature^46^:

19 $$\alpha { = }\frac{{{\text{lg}}\left( {{\text{m}}_{{\text{r}}} {\text{/m}}} \right)}}{{{\text{lgr}}}}$$

where: m_r_ is the mass of the fragment whose particle size is smaller than r, and m is the total mass of the sample fragment.

20 $${\text{D}} = {3} - {\upalpha }$$

where: α is the slope in a log–log coordinate system formed by lg(m_r_/m) and lgr:

After testing, the crushed coal samples are screened through round-hole coal screens with diameters of 30 mm, 20 mm, 10 mm, 3 mm, 2 mm and 1 mm. The round-hole coal screens are shown in Fig. 17. The coal samples below the diameter of each grade are weighed as shown in Fig. 18, and then the fractal dimension of the coal samples under different conditions can be calculated using formulas (19), (20). Table 3 contains the parameters.

**Table 3:** Fractal analysis of coal sample fragmentation.

Coal sample ID	Sieve diameter/mm						Gross mass	α	Fractal dimension	Energy absorption density/J cm^-3^
	30	20	10	3	2	1				
A-1	58.71	55.39	49.30	28.53	23.74	14.02	77.21	0.409	2.591	3.091
A-2	26.70	24.25	15.57	7.51	5.85	3.56	79.83	0.602	2.398	2.466
A-3	40.73	31.77	20.16	9.85	8.59	4.43	79.11	0.625	2.375	3.883
A-4	10.16	5.74	5.09	2.63	2.02	1.05	76.71	0.594	2.406	4.056
A-5	39.63	33.10	22.00	9.98	7.36	4.27	77.76	0.655	2.345	3.085
B-1	1.16	1.16	1.16	0.83	0.77	0.48	83.29	0.239	2.361	3.511
B-2	16.23	10.69	9.26	5.43	4.31	2.31	84.92	0.510	2.490	7.464
B-3	65.68	49.95	32.24	18.46	15.10	8.69	78.85	0.564	2.436	17.012
B-4	55.99	42.91	35.74	21.54	16.15	9.58	76.92	0.483	2.517	19.451
B-5	80.46	72.81	52.02	33.92	30.00	20.53	80.46	0.395	2.605	32.356
C-1	66.86	50.32	38.78	22.65	17.76	10.99	78.24	0.501	2.499	2.392
C-2	28.75	20.49	10.64	4.71	3.97	2.43	82.81	0.720	2.380	2.660
C-3	31.86	22.64	11.74	6.60	5.56	3.21	79.57	0.642	2.358	3.091
C-4	12.60	10.48	7.83	4.33	3.25	1.88	78.63	0.540	2.260	3.075
C-5	5.14	0.18	0.18	0.03	0.03	0.03	79.36	1.277	1.723	7.498

**Figure 19:**
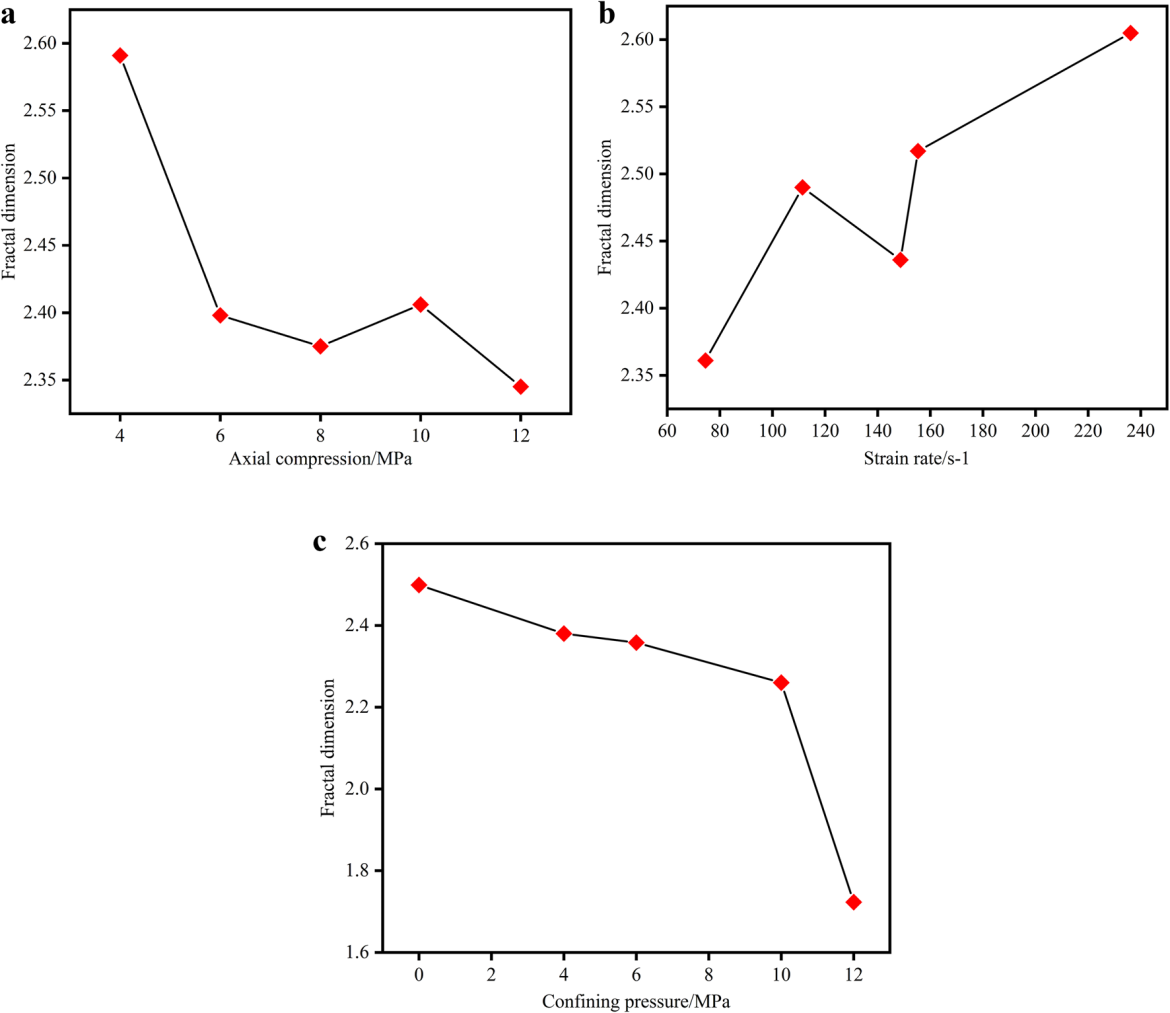
Axial pressure (**a**) strain rate (**b**) confining pressure (**c**) vs. fractal dimension.

In the relationship between axial pressure and fractal dimension, Fig. 19a, it can be seen that, except for the data at the point where the axial pressure is 12 MPa, the fractal dimension first decreases and then increases as the axial pressure increases. This trend is due to the following reasons: When the axial pressure is less than 8 MPa, the axial pressure compresses the internal cracks in the coal samples, which leads to an increase in the dynamic strength of the samples in the process, and the coal samples are not so easy to crush after being subjected to the impact load. When the axial pressure is more than 8 MPa, the relatively high axial pressure promotes the expansion of the cracks, so the coal specimens are more easily broken under the impact load. Therefore, under this condition, the fractal dimension increases with the increase of axial pressure.

The fractal dimension of the coal specimens at different strain rates is plotted against the strain rate. The relationship between the fractal dimension of the coal specimens and the strain rate is shown in Fig. 19b. From this, it can be seen that the fractal dimension of the coal specimens under the three-dimensional dynamic and static combined loading increases with increasing strain rate, except for the data at a strain rate of 148.645/s, and that the coal specimens are more susceptible to severe impacts due to the increase in strain rate. The higher the strain rate is, the more likely the coal sample is to break and deform as a result of a severe impact, and the smaller the broken blocks of the coal sample are, which leads to an increase in the fractal dimension of the coal sample with the increase of the strain rate.

From Fig. 19c, it can be seen that the fractal dimension of the coal specimens decreases with the increase of confining pressure, which can be analyzed in Chapter 2, and the existence of peripheral pressure prevents the damage of the coal specimens in the confining direction, resulting in an increase in the dynamic strength of the coal specimens with the increase of confining pressure, similarly, under the same impact velocity and axial pressure, the crushing degree of the coal sample decreases with the increase of the confining pressure, and on the contrary, the crushing degree of the coal block increases with the increase of the confining pressure, so that under the same conditions, the crushing degree of the coal sample increases with the increase of the confining pressure. Under other conditions, the fractal dimension of the coal sample decreases with the increase of the confining pressure.

## Conclusion

With the expansion of mineral resource extraction to depth, deep coal rock is subject to three-dimensional prestatic load stresses and severe disturbance of these complex storage conditions, often resulting in ground pressures, rock explosions, road collapses and other dynamic disasters. In order to provide theoretical guidance to prevent the above power disasters, the triaxial Hopkinson test facility (SHPB) is used in this paper to carry out power impact tests on coal samples under different loading conditions and analyze the change rule of their mechanical properties. Based on the three-dimensional dynamic and static combined loading test, the destruction mode of coal specimens and the change rule of fractal dimensionality are discussed. ANSYS/LS-DYNA numerical simulation software is used to simulate the testing process of coal samples under three-dimensional dynamic and static combined loading, and numerical simulation tests are used to verify the accuracy of the indoor tests and theoretical analysis results.

1. The stress–strain curves of the coal specimens under different strain rates, confining pressures and axial pressures basically have the same trend, and the stress–strain curves of the three-dimensional dynamic-static combined impact test are different from those under conventional impact conditions. The stress–strain curves of the coal samples under different strain rates, different confining pressures and axial pressures all showed a double-peak phenomenon. The curves show a certain jumpiness, especially the most obvious double-peak phenomenon at different strain rates. The reason for this phenomenon could be related to the role of charcoal in micro fracturing the crystals.
2. The peak dynamic stress and the section modulus of coal samples both increase linearly with the increase in strain rate and also linearly with the increase in peripheral pressure. Under the impact loading, the peripheral pressure restricts the expansion of the internal cracks in the coal rock, so the peak dynamic stress of the coal rock under three-dimensional dynamic and static combined loading increases with the increase of the peripheral pressure and decreases with the increase of the axial pressure. This basically shows the relationship between the change of one-dimensional quadratic equation. The dynamic peak stress of coal rock under combined load is basically a quadratic equation.
3. In the three-dimensional dynamic-static combination loading, the degree of crushing of coal samples increases with the increase of strain rate, decreases with the increase of peripheral pressure, and first decreases and then increases with the increase of axial pressure. The damage modes of the coal specimens under the three-dimensional dynamic-static combination loading were mainly “X”-type and “conical” shear damage modes with a small amount of tensile damage. At the same time, the fractal dimension increases with the increase of strain rate, decreases with the increase of peripheral pressure, and decreases and increases again with the increase of axial pressure.
4. The maximum relative errors between the simulation results and the experimental data are (Group A: 2.9578%, Group B: 6.177%, Group C: 6.382%), indicating that the dynamic performance of the coal specimens in the three-dimensional impact test can be better simulated by the construction of the HJC model.

## Data Availability

The datasets used and/or analysed during the current study are available from the corresponding author on reasonable request.
